# Revealing reaction intermediates in one-carbon elongation by thiamine diphosphate/CoA-dependent enzyme family

**DOI:** 10.1038/s42004-024-01242-y

**Published:** 2024-07-21

**Authors:** Youngchang Kim, Seung Hwan Lee, Priyanka Gade, Maren Nattermann, Natalia Maltseva, Michael Endres, Jing Chen, Philipp Wichmann, Yang Hu, Daniel G. Marchal, Yasuo Yoshikuni, Tobias J. Erb, Ramon Gonzalez, Karolina Michalska, Andrzej Joachimiak

**Affiliations:** 1 eBERlight and Structural Biology Center, X-ray Science Division, Argonne National Laboratory, Lemont, IL USA; 2 Consortium for Advanced Science and Engineering, University of Chicago, Chicago, IL USA; 3 Department of Chemical, Biological, and Materials Engineering, University of South Florida, Tampa, FL USA; 4 Department of Biochemistry and Synthetic Metabolism, Max Planck Institute for Terrestrial Microbiology, Marburg, Germany; 5 The US Department of Energy Joint Genome Institute, Lawrence Berkeley National Laboratory, Berkeley, CA USA; 6 Center for Synthetic Microbiology (SYNMIKRO), Marburg, Germany; 7 Department of Biochemistry and Molecular Biology, University of Chicago, Chicago, IL USA

**Keywords:** X-ray crystallography, Enzyme mechanisms, Biocatalysis

## Abstract

2-Hydroxyacyl-CoA lyase/synthase (HACL/S) is a thiamine diphosphate (ThDP)-dependent versatile enzyme originally discovered in the mammalian α-oxidation pathway. HACL/S natively cleaves 2-hydroxyacyl-CoAs and, in its reverse direction, condenses formyl-CoA with aldehydes or ketones. The one-carbon elongation biochemistry based on HACL/S has enabled the use of molecules derived from greenhouse gases as biomanufacturing feedstocks. We investigated several HACL/S family members with high activity in the condensation of formyl-CoA and aldehydes, and distinct chain-length specificities and kinetic parameters. Our analysis revealed the structures of enzymes in complex with acyl-CoA substrates and products, several covalent intermediates, bound ThDP and ADP, as well as the C-terminal active site region. One of these observed states corresponds to the intermediary α–carbanion with hydroxymethyl-CoA covalently attached to ThDP. This research distinguishes HACL/S from related sub-families and identifies key residues involved in substrate binding and catalysis. These findings expand our knowledge of acyloin-condensation biochemistry and offer attractive prospects for biocatalysis using carbon elongation.

## Abstract (web summary)

2-Hydroxyacyl-CoA lyase/synthase (HACL/S) is known to catalyze the condensation of formyl-CoA with aldehydes or ketones, however, the mechanism of one-carbon elongation biochemistry is not well understood. Here, the authors report the structures of enzymes in complex with co-factors, substrates, and products, revealing key intermediates and the C-terminal active site region, and distinguishing them from related sub-families.

## Introduction

Thiamin diphosphate (ThDP) is a critical cofactor for many biochemical transformations across all kingdoms of life^1,2^. ThDP aids in making and breaking bonds between carbon and other elements including sulfur, oxygen, hydrogen, or nitrogen and, remarkably, even between two carbon atoms. Given their versatility, ThDP-dependent enzymes have recently garnered significant interest as tools for biotransformation.

One such enzyme is 2-hydroxyacyl-CoA lyase/synthase (HACL/S), which catalyzes the reversible cleavage of 2-hydroxyacyl-CoA (2hcCoA) into formyl-CoA (fCoA) and an aldehyde or ketone. Originally identified in the mammalian α-oxidation pathway of 3-methyl-branched fatty acids^3^, HACL/S has demonstrated remarkable catalytic reversibility, as it is capable of condensing fCoA with various substrates covering a wide range of aldehydes and ketones with different carbon chain lengths^4–8^. Recently, the importance of HACL/S in microbial and biochemical conversion of one-carbon (C1) compounds to value-added products has been highlighted, leveraging the wide substrate specificity range of the enzyme for iterative C1 elongation to yield a variety of small molecules^7^. Additionally, HACL/S-based pathways are favored for synthetic biology applications due to their superior kinetic parameters compared to C1 assimilation by other ThDP-dependent carboligases^6,9^. Although HACL/Ss have been the focus of several biochemical studies, and some structural analysis was performed based on homology-guided modeling, most studies have centered on the acyloin condensation reaction between formaldehyde and fCoA (C1-C1 condensation)^4–6^. However, several HACL/S variants have shown broader substrate specificity, with the conversion of longer-chain aldehydes expanding the range of potential products^4,5,10^. For HACL/S-based pathways to be fully exploitable, an understanding of its ligand specificity and molecular-level catalytic mechanisms, as well as the relationship between sequence, structure, and catalytic function, is necessary. Unfortunately, the lack of high-resolution structural data has so far hindered such analyses.

Crystal structures of oxalyl-CoA decarboxylases (OXC) from various procaryotic species^5,11–13^ and 2-hydroxyisobutyryl-CoA lyases/synthases (HICL/S) from *Actinomycetospora chiangmaiensis* DSM 45062 (AcHICL/S, PDB ids: 7pt1-4^14^), known to be related to HACL/S enzymes both phylogenetically and structurally, are available and their catalytic mechanisms have been elucidated. In these sub-families, the mechanism is similar and reactions proceed via a ThDP-2-hydroxymethyl-CoA (ThDP-hmCoA) intermediate with α-carbanion/enamine resonance structure, resulting from the departure of an aldehyde from 2-hydroxyacyl-CoA or decarboxylation of oxalyl-CoA (oCoA)^5^. However, there are clear differences in their substrate specificities, with HACL/S favoring aldehydes, HICL/S ketones and OXC CO_2_. With its preference for aldehydes, HACL/S is the most promising candidate for bioproduction cascades, particularly those relying on iterative C1 elongation^7^. However, past mutagenesis studies relied on structures without bound cofactor or substrate and a disordered active site covering loop (PDB id: 6xn8) or on low accuracy AlphaFold2^15^ predictions based on a limited number of high-resolution crystal structures of HICL/S, OXC and HACL/S. The HICL/S structure in particular serves as a poor model for HACL/S, as it is a more distant relative and diverges significantly in structure, lacking an ADP-binding domain and containing a distinct, significantly longer C-terminal active site covering region^14^. While OXC is closer related, beneficial active site mutations acquired in it do not provide benefit to HACL/S^5^, and OXC itself cannot be remodeled to HACL/S-like activity based on existing structures^4,5^. In the case of HACL/S, the only available structure is that of RuHACL/S (PDB id: 6xn8), but it is missing key ligands (substrate and product acyl-CoAs) and its C-terminal covering segment is disordered, which in related enzymes plays an important function in ligand binding, recognition and catalysis^12,14^. This reveals a need for high-quality HACL/S structures resolving the full enzyme, cofactors and substrates to give better insight into the catalytic mechanism and the structure of the active site.

Using a multiple sequence alignment with structure modeling of homologs, we have recently identified key ligand interactions and catalytic regions such as the ThDP and ADP-binding, CoA-binding and C-terminal regions, and conserved residues among enzymes exhibiting condensation activities between formaldehyde and fCoA. However, despite considerable progress in computational tools such as AlphaFold2^15^, very few high-resolution crystal structures of related enzymes are available in the Protein Data Bank (PDB) to provide the necessary atomic-level accuracy. Therefore, further progress in elucidating experimental protein structures in complexes with ligands (substrates, cofactors, and products) is essential in understanding the roles of specific amino acid residues in binding and catalysis.

Here we present the crystal structures of five members of the HACL/S sub-family in complex with ThDP and ADP. We were able to obtain structures of two enzymes that in addition to ThDP and ADP contain a substrate/cofactor (fCoA) and products (2-hydroxyisobutyryl-CoA (hCoA), D-lactyl-CoA (D-lCoA), and L-lactyl-CoA (L-lCoA)). These structures mark the first instances of a fully ordered C-terminal active site covering region resolved in the HACL/S sub-family. For this study, the HACL/S enzymes were selected based on relative synthase activity and substrate specificity, covering aldehydes with different chain lengths. Structural analysis of the variants highlights remarkable characteristics of HACL/S sub-family enzymes that distinguish them from the OXC and HICL/S sub-families. The study of HACL/S sub-family enzymes with various catalytic efficiencies and substrate preferences, in terms of their mechanistic and structural aspects, enhances our knowledge of acyloin-condensation biochemistry and offers exiting prospects for biocatalysis. Considering HACL/Ss as C1 elongation enzymes is particularly interesting, as it can be further extended to diverse bioproduct synthetic pathways^7^.

## Results

**Fig. 1:**
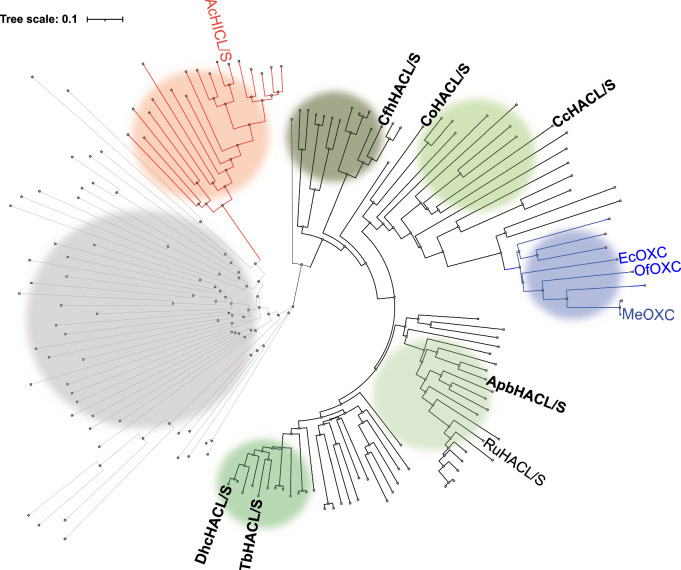
Phylogenetic tree of the 2-hydroxyacyl-CoA lyase/synthases (HACL/S) sub-families (colored circles). Catalytically active HACL/S sub-families (green circles with varying darkness), oxalyl-CoA decarboxylase (OXC) (blue), 2-hydroxyisobutyryl-CoA lyases/synthases (HICL/S) (red) and inactive HACL/S homologs (gray) synthesized and screened for HACL/S activities. HICL/S (*Actinomycetospora chiangmaiensis* DSM 45062 (AcHICL/S)) and OXC (*E. coli* (Ec), *Oxalobacter formingenes* (Of) and *Methylorubrum xtorquens* (Me)OXC) sub-families include enzymes with crystal structures available. Enzyme names highlighted in bold have been investigated in this work.

In our previous study, using gene homolog bioprospecting and clustering based on sequence similarity, we identified, produced and screened 134 ThDP-dependent enzyme homologs for HACL/S condensation activities^6^. A phylogenetic tree built on these variants revealed one large clade, a smaller clade, and other dispersed branches with relatively low sequence similarities (Fig. 1). The large clade harbors multiple HACL/S enzyme sub-families with catalytically active variants, which share common features, such as an ADP-binding domain (Fig. 1, green branches). Among them, there is a branch containing known OXC enzymes, which we therefore define as the OXC sub-family (Fig. 1, blue branches). The HICL/S sub-family, containing AcHICL/S, is more distantly related to HACL/S (Fig. 1, red branches). A last clade (Fig. 1, light gray branches) harbors enzymes with sequence similarity to HACL/S enzymes but no activities with the substrates were tested and there is no predicted specific biochemical function(s).

For this study, we carefully chose six variants of the HACL/S enzyme that exhibited high condensation activity with C1–C3 aldehyde compounds and formyl-CoA. We also made sure that the variants had diverse amino acid sequences to broadly cover the HACL/S sub-family and were produced well in *Escherichia coli* (Fig. 1, Supplementary Fig. S1). HACL/S enzymes highlighted in bold in Fig. 1 represent HACL/S variants investigated in this work.

### Overview of crystal structures

**Fig. 2:**
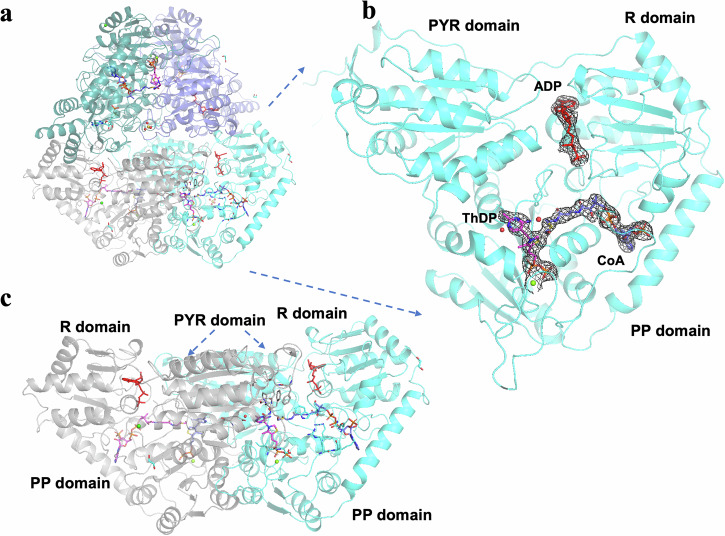
Overall structure of *Alphaproteobacteria bacterium* 2-hydroxyacyl-CoA lyase/synthase (ApbHACL/S) dimer in complex with CoA and ADP. Structure is shown in two orthogonal orientations (**a**, **c**). **b** shows HACL/S domains with ThDP (magenta), ADP (red) and CoA (blue). The electron density (composite annealed map, 2DFo-mFc) for the ligands is contoured at 1.0 σ level.

Using synthetic gene technology optimized for expression in *E. coli*, we produced and purified six recombinant enzymes, which were characterized biochemically. We crystallized and determined structures of five members of the HACL/S sub-family (ApbHACL/S, DhcHACL/S, CfhHACL/S, CcHACL/S and TbHACL/S (Fig. 1), (see Material and Methods for enzyme name designation). For the sixth variant, CoHACL/S, we were unable to obtain well diffracting crystals. Crystal structures for apoenzymes and complexes with acyl-CoA substrates, ThDP cofactor, ADP, and with products were determined at high resolution (1.70–2.70 Å) (Supplementary Table S1). All crystal structures were determined by molecular replacement using AlphaFold2 models as search templates. All the enzymes are tetramers, specifically dimers of dimers (α_2_)_2_, where α_2_ dimer represents a catalytical unit. The α_2_ interface hosts the ligand binding and catalytic sites that are well-conserved among ThDP-dependent lyases (Fig. 2, Supplementary Fig. S1). The overall structural analysis is based on highest resolution structures (1.70 Å TbHACL/S and 1.85 Å CcHACL/S), unless indicated otherwise.

Like in other ThDP dependent enzymes, the protomer consists of three domains with similar α/β/α fold. Each domain is composed of a twisted β–sheet containing six parallel β–strands surrounded by three α-helices on each side of the sheet. The three domains have specific functions: the N-terminal domain binds the pyrimidine of ThDP (PYR), the middle domain has a regulatory function (R) and binds ADP, and the C-terminal domain binds the pyrophosphoryl group (PP) of ThDP (Fig. 2). The R and the PP domains are connected by a long α-helix. The dimer interface includes three parts: one produced between the two symmetrically related PYR domains interacting mainly through α-helices, second formed between the two PP domains, and third created between the PYR domain from one protomer and the PP domain from the second protomer. The cofactor ThDP resides at the PYR/PP dimer interface, the pyrimidine moiety binds to the PYR domain from one protomer and the pyrophosphoryl moiety binds to the PP domain from the other protomer (Fig. 2). Acyl-CoA substrates bind in the ridge between the PP and R domains and the ADP ligand lies in the crack formed between the PYR domain and the middle domain of the same protein chain (Fig. 2).

We observed different structural features among the variants. In all ApbHACL/S structures, the whole tetramer is in the asymmetric unit. The monomers are structurally very similar to each other, with root mean square deviation (RMSD) on superimposed Cα positions in the range of 0.2–0.4 Å (Supplementary Fig. S2) but locally show distinct conformations. Therefore, there are four different representations of the active sites formed by four slightly altered protomers in the tetramer. Each structure revealed unique disordering of the acyl moiety of the CoA compound, the presence or absence of water molecules and other ligands produced during the catalytic reaction, such as acetaldehyde, including the CoA sulfur atom for some CoA compounds (Supplementary Fig. S3). On the other hand, the asymmetric units of CoA compound-TbHACL/S complex contain only two slightly different monomers (protein chains A and B), each protomer from two separate catalytic pairs (AA’ and BB’, a prime indicates symmetry related partner), which means each catalytic dimer is formed by the structurally identical monomers. However, in the tetramer, the two dimers are slightly different, but contain identical catalytic sites. The active sites of all the enzymes showed a similar conformation regardless of the CoA ligand’s presence (see below).

### Sequence and structure comparison of HACL/S enzymes to related sub-families

As expected, comparative sequence and structure analysis demonstrated a close relationship of the chosen HACL/S sub-family members and RuHACL/S (PDB id: 6xn8). Sequence identities ranged 40–62% and RMSDs for superimposed Cα atom positions per ~540 atoms in the range of 0.73–1.25 Å (with the C-terminal tail residues excluded) (Supplementary Table S2, Supplementary Figs. S1 and S2). In a similar vein, the HACL/S enzymes showed good similarity to OfOXC (PDB ids: 2ji8, 2jib) and EcOXC (PDB id: 2q27), with sequence similarity ranging from 37-42% and RMSDs in the range of 1.23–1.60 Å (Supplementary Table S2).

Finally, AcHICL/S (PDB id: 7pt4) showed the biggest divergence from the HACL/S variants (sequence similarity 23–29%, RMDS 1.71–1.92 Å). The major difference between RuHACL/S and AcHICL/S, except for the first ~15 N-terminal residues, stems from the α-helix spanning residues N491 - N500 which is followed by the loop (Y501-D513). In HACL/S and OXC enzymes, this region is a loop, and a part of long loop (N472-Y499 in ApbHACL/S) between a β-strand and an α-helix which also contains an I475-G476 motif (in ApbHACL/S), with the main chain nitrogen atom of Gly476 interacting with the pyrophosphoryl group of ThDP. This α-helix in AcHICL/S stays close to the protein body, however, the loops without this α-helix tend to swing out from the protein bodies in all other enzymes. Moreover, differences in key residues in R domain of AcHICL/S explain why members of this sub-family do not bind ADP^14^.

When the positions of the Cα atoms of the active site residues and four Arg residues in ApbHACL/S (R156 and R276 involved in ADP binding and R260 and R400 binding acyl-CoA) are compared, the RMSDs with AcHICL/S residues are considerably higher (1.37–1.52 Å) than among HACL/S (0.20–0.80 Å) (Table 1). When Arg side chains are removed from the comparison, the RMSDs with AcHICL/S are still high.

These structural comparisons indicate that HACL/S and OXC enzymes have more similar active sites and are quite different from that of AcHICL/S, consistent with phylogenetic analysis (Fig. 1). The high RMSDs between HACL/Ss and AcHICL/S perhaps reflect the lack of an ADP binding site in the R domain of AcHICL/S. These differences in structure and active site composition likely contribute to distinct activity profiles and some mechanistic differences among these enzymes (for example HACL/S vs OXC enzymes) sub-families and support the need for high-quality HACL/S structures for HACL/S engineering.

### Active site comparison between HACL/S variants

**Fig. 3:**
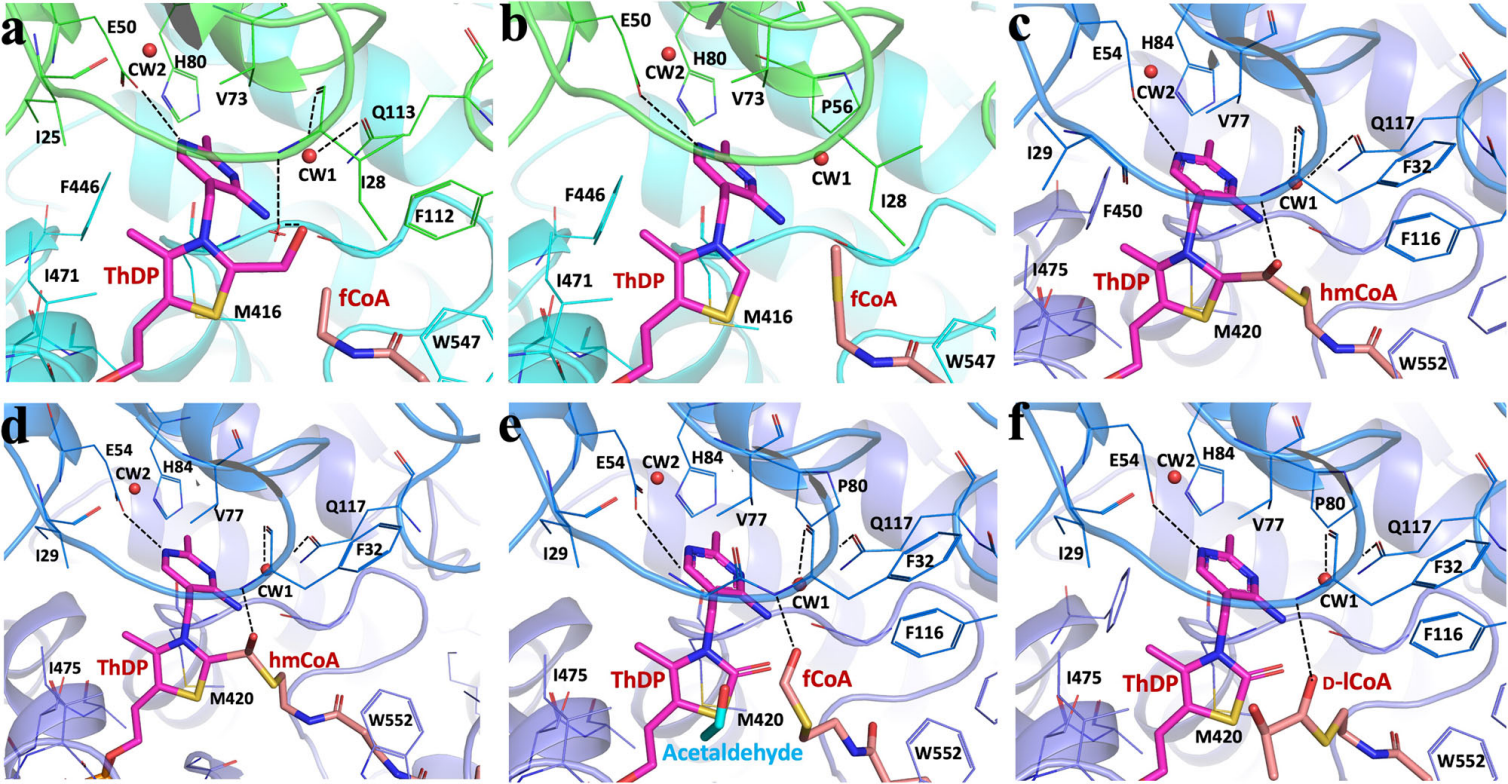
Active sites of *Thermoflexaceae bacterium* 2-hydroxyacyl-CoA lyase/synthases (TbHACL/S) and *Alphaproteobacteria bacterium* HACL/S with ThDP and CoA derivatives. **a** TbHACL/S-formyl CoA (fCoA) complex, **b** TbHACL/S-2-hydroxyisobutyryl CoA (hCoA) complex, **c** ApbHACL/S-CoA complex, **d** ApbHACL/S-fCoA complex, **e** ApbHACL/S-L-Lactyl CoA (lCoA) complex and **f** ApbHACL/S-D-lCoA complex. TbHACL/S dimer is in cyan/dark cyan, ApbHACL/S dimer in dark blue/blue, ThDP and CoA compounds are depicted with magenta and orange sticks. Electron density for whole ligands in these structures are shown in Supplementary Fig. S3.

Our structures of HACL/S enzymes reveal a very well-defined active site with excellent electron density for ThDP, ADP, the majority of acyl-CoA, and the side chains of active site residues (Fig. 3, Supplementary Figs. S3 and S4).

**Fig. 4:**
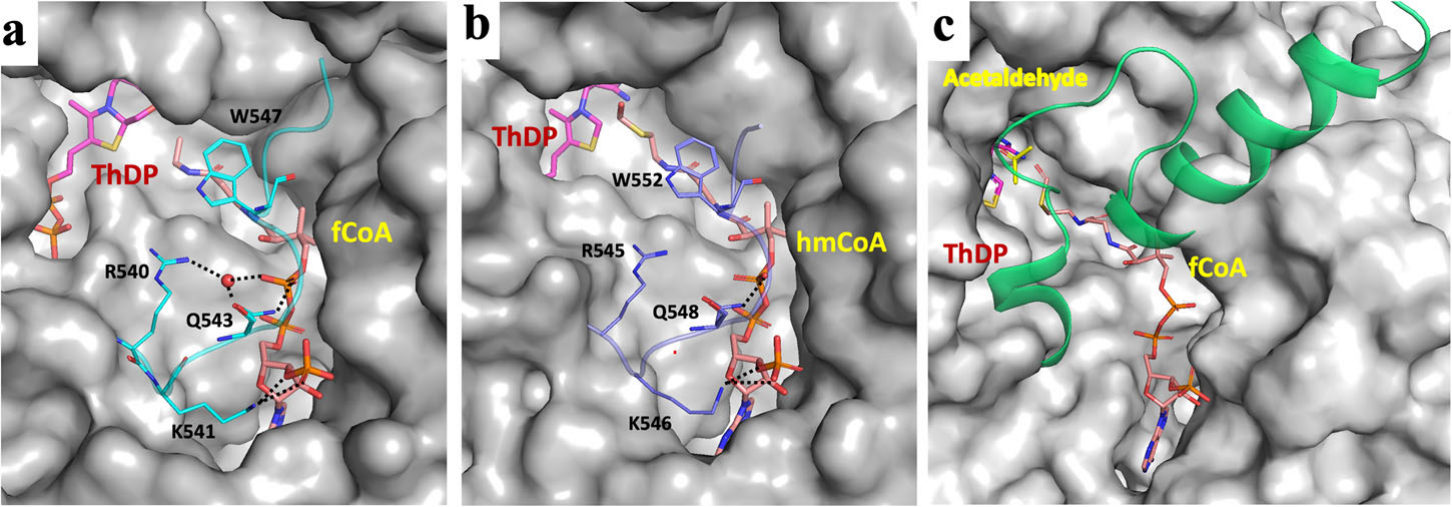
Ordered C-terminal peptides covering active site of HACL/S enzymes. **a** *Thermoflexaceae bacterium* 2-hydroxyacyl-CoA lyase/synthases (TbHACL/S) (PDB id: 8vze), **b** *Alphaproteobacteria bacterium* HACL/S (PDB id: 8vfc), and **c** *Actinomycetospora chiangmaiensis* DSM 45062 2-hydroxyisobutyryl-CoA lyases/synthases (HICL/S) (from PDB id: 7pt4)^14^ with ligands bound in the active site.

From crystallography of OfOXC^12^ and AcHICL/S^14^, it was demonstrated that the C-terminal covering peptide can be resolved only when the enzyme is saturated with acyl-CoA, as the region works as a lid folding over the CoA binding domain, likely to stabilize the acyl-CoA-enzyme complex^14^. Based on these results, we attempted to saturate the binding sites with ligands (see Materials and Methods). These efforts were successful for two our enzymes, where an ordered C-terminal region could be observed (Fig. 4).

A total of six structures of TbHACL/S and ApbHACL/S in complex with acyl-CoA showed well-ordered C-terminal peptide (two representative examples are shown in Fig. 4a, b). The conformation of this region is very different from AcHICL/S (Fig. 4c). Several residues engage in close contacts with acyl-CoA (R545, K546, Q548, and W552 in ApbHACL/S and R540, K541, Q543 and W547 in TbHACL/S) through electrostatic, hydrogen bonds and van der Waals interactions. The C-terminal covering regions are coming from the second protomer (Fig. 4, Supplementary Table S3). These contacts seem stabilizing and augmenting cofactor interactions with the active site, likely extending the “on” state of the acyl-CoA.

Due to the high structural similarity and amino acid conservation of the active sites (Fig. S2, Supplementary Table S2), the catalytically important regions will be discussed on the basis of AbpHACL/S (Figs. 3 and 4). In variants with bound substrate and/or product and a fully ordered C-terminal covering peptide, the canonical catalytic glutamic acid residue (E50) forms a strong hydrogen bond (2.60–2.90 Å) between the carboxylate oxygen of Glu and N1’ of ThDP (Fig. 3, Supplementary Fig. S4, Table S3). This interaction is critical for the activation of ThDP.

The conserved water molecule between N4’ and C2 of ThDP is not observed in any of our structures, however, in some structures, there is strong electron density nearby that substrates and products can be modeled into (see below). Residues F-E (116-117) and the loop containing motif G-I-P (31-33), involved with the $\alpha$-carbanion/enamine intermediate^12^, are from one protomer while the residues directly binding the ThDP, acyl-CoAs and ADP are from the complementary unit. More interactions with ligands are provided by main chain atoms in the loop I475-G476 binding the pyrophosphoryl moieties of ThDP, and two arginine residues (R260 and R400) binding the acyl-CoAs. Two additional arginine residues (R156/R276) interact with ADP. All these residues are from the second protomer and are conserved in HACL/Ss.

In some crystals supplied with acyl-CoA, we could observe a reaction occurring (Fig. 3, Supplementary Fig. S3). Here we note that enzymes were co-crystallized with acyl-CoA, giving them a minimum of three days to react before cryo-protection and data collection. Of particular interest here are the structures of ApbHACL/S with fCoA and D-/L-lCoA (Fig. 3d–f).

**Fig. 5:**
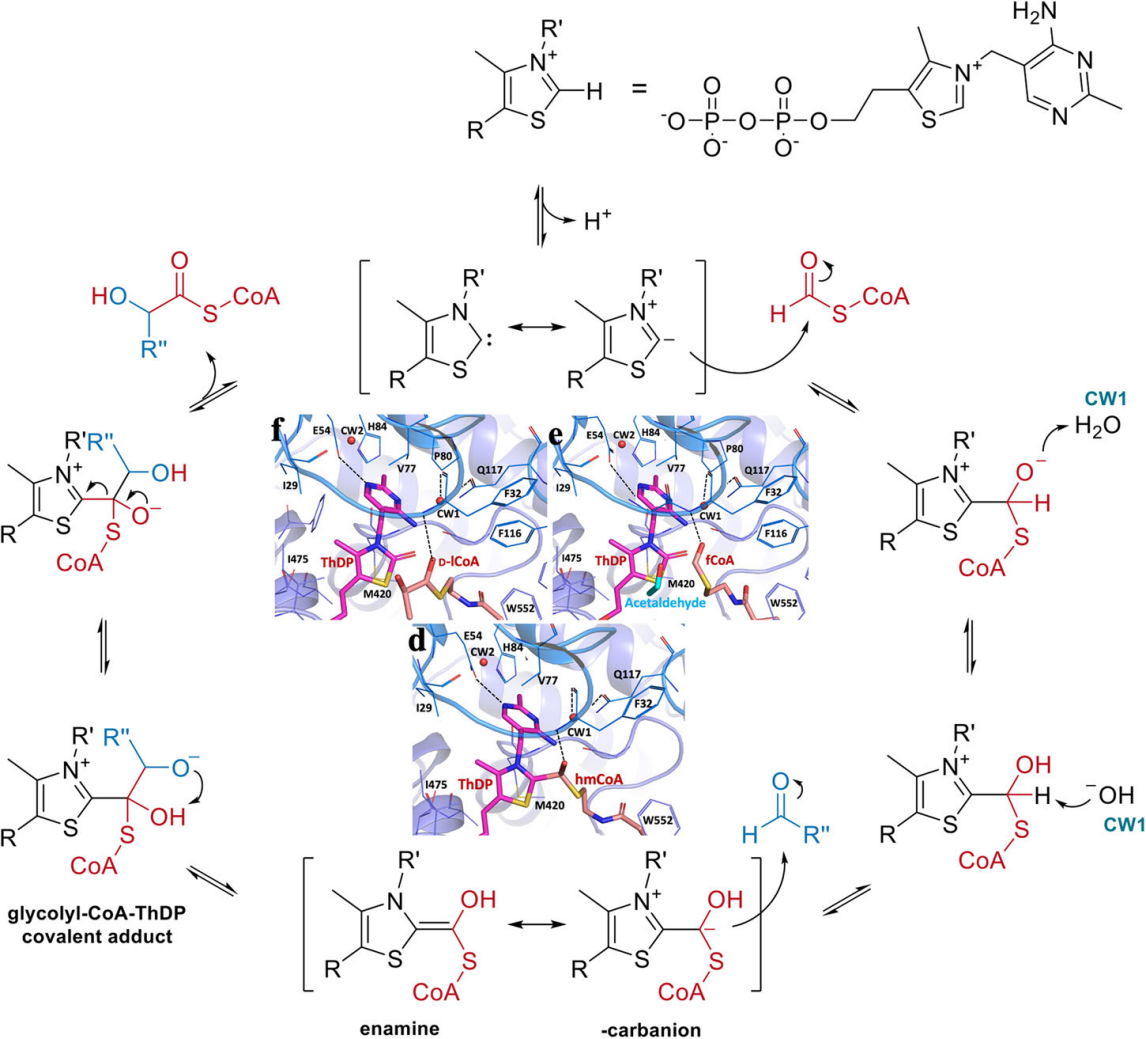
Mechanism of the synthetic reaction supported by enzymes from oxalyl-CoA decarboxylase (OXC), *Rhodospirillales bacterium* URHD0017 2-hydroxyacyl lyase/synthase (HACL/S) and *Actinomycetospora chiangmaiensis* DSM 45062 2-hydroxyisobutyryl lyase/synthase (HICL/S) sub-families. This canonical pathway of C1 addition to aldehydes by HACL/S was adapted from Nattermann, Burgener et al., 2021^5^. The pictures of α-carbanion intermediate, product and substrate formed during the *Alphaproteobacteria bacterium* HACL/S catalysis reactions with substrates formyl CoA (d), L-lCoA (**e**) and D-lCoA (f) are inserted in the middle.

In the structure of ApbHACL/S incubated with fCoA, we trapped a covalent intermediate corresponding to ThDP-hmCoA. This adduct is a product of deprotonated ThDP reacting with fCoA (Fig. 5). A similar intermediate ThDP-hmCoA is also found in the structure of ApbHACL/S-CoA. In these two structures ApbHACACL/S-CoA and ApbHACL/S-fCoA, the distances between C2 of ThDP and C1 of hmCoA are significantly longer than a regular C–C bond (1.54 Å) with 1.8 and 1.9 Å, respectively. Additionally, the C1-O distance in ApbHACL/S-CoA is 1.4 Å which is close to that of a regular hydroxymethyl but 2.0 Å for the same distance in ApbHACL/S-fCoA is much elongated. This suggests that these two intermediates found in these structures represent slightly different reaction states.

We identified two water molecules (CW1 and CW2 indicated in Fig. 3) in the middle of the catalytic center that are conserved among all structures (except for the DhcHACL/S due to a relatively low resolution of 2.70 Å). These molecules are in position to be activated for catalysis. CW1 is located near the Glu/Gln side chain (Q113 in DhcHACL/S (PDB id: 8vzj) and TbHACL/S (PDB id: 8vzh) and D117 in ApbHACL/S (PDB id: 8vzf), close to the mainchain carbonyl oxygen atom of F32 in ApbHACL/S or I28 in TbHACL/S (2.9 Å), oxygen atom of D117 or Q113 (3.0 Å), and the acyl group of CoA derivatives when they can be modeled in the structures (3.2 Å) (Fig. 3). The second conserved water molecule, CW2, is further away from the catalytic center but close (2.6–2.9 Å) to the carboxylate oxygen atom of catalytic residue Glu50/54 which activates C2 of ThDP via interacting with N1 of ThDP (Fig. 3).

In some structures we observe products of reactions that seem off pathway. In the TbHACL/S-hCoA complex structure there is a covalent adduct of hydroxymethyl linked to the ThDP thiazolium ring C2 carbon (Fig. 3a, Supplementary Fig. S5).

The oxygen atom is out of plane of the thiazolium ring C2—hydroxymethyl carbon plane suggesting single C–C bond and single C–O bond (“hydroxymethyl-ThDP”, Supplementary Fig. S7) (PDB id: 8vze). In both cases, the C2-C distance is elongated to 1.7–1.9 Å and the C–O bond remains 1.4 Å. In the structures of complexes ApbHACL/S-L-lCoA (Fig. 3e) and ApbHACL/S-D-lCoA (Fig. 3f), there is an oxygen atom attached to thiazolium C2, suggesting the presence of a carbonyl bond (C2 = O) with a distance of 1.3 Å (“oxo-ThDP”, Supplementary Fig. S7) (PDB ids: 8vza, 8vzb). In these two structures, the phosphopantetheine chain of the acyl-CoA is ordered, with the product visible in electron density attached to CoA. The products can be explained by reaction of ThDP with formaldehyde^2^ or reactive oxygen species (ROS). In Supplementary Fig. S7, we provide possible mechanisms of reactions leading to these off-pathway products. Formation of such adducts will lower overall yield of the anticipated condensation reaction product. Further investigation of these reactions combined with mutagenesis may allow improvement of the pathway.

**Fig. 6:**
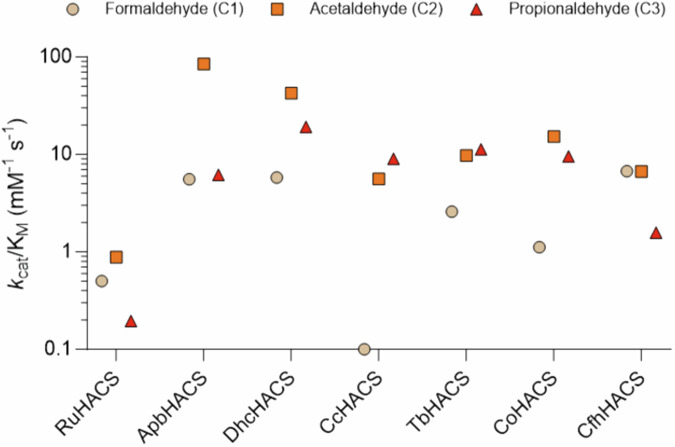
Dot plot summarizing catalytic efficiencies (*k*_cat_/*K*_M_) of 2-hydroxyacyl-CoA lyase/synthases (HACL/S) variants demonstrating substrate (chain length) specificity. Full kinetic parameters can be found in Supplementary Table S2. *Rhodospirillales bacterium* URHD0017 HACL/S was chosen as a reference.

We have determined kinetic parameters for purified HACL/S (Fig. 6 and Supplementary Table S4). All tested HACL/S showed reasonable activity on two carbon long (C2) substrates, which are mostly preferred over both C1 and C3. The most unique variant is CcHACL/S, showing a clear preference for C3 over C2, and no activity on C1 substrate at all (Fig. 6, Supplementary Table S4). C1 substrate may be too small to interact with this surface. Curiously, this variant clusters in close proximity with the OXC sub-family (Fig. 1), which shows clear preference on C1 and C2 over C3 aldehydes^10^. Nevertheless, these enzymes show distinct distributions of specificities and different kinetic rates for different substrates. For example, RuHACL/S is at least an order of magnitude slower than other tested enzymes. It uniquely has Pro493 residue in the active site, making it more rigid and therefore less active.

## Discussion

We have determined ten high-resolution structures of enzymes from the HACL/S sub-family, including DhcHACL/S, CcHACL/S, TbHACL/S, CfhHACL/S, and ApbHACL/S and measure kinetic parameters for all purified enzymes. All structures contain well-defined bound ThDP and ADP. In addition, six structures contain CoA derivatives and products of the reaction. ApbHACL/S yielded the structures with CoA-ThDP and ThDP-hmCoA, as well as complexes with acetaldehyde, fCoA, L-lCoA and D-lCoA. TbHACL/S was co-crystallized in the presence of fCoA and hCoA. Five structures contain on and off pathway intermediates (Fig. 3a, c–f). In several structures CoA cofactors are well ordered but in some the tail sections are partly disordered, including the sulfur atom and acyl moiety (Supplementary Fig. S3). Six structures with bound acyl-CoA molecules show C-terminal regions ordered, four for ApbHACL/S and two for TbHACL/S (Fig. 4, PDB ids: 8vzf, 8vzc, 8vza, 8vzb, 8vzd, 8vze).

Our structures provide insights into the generally accepted catalytic mechanism of HACL/S enzymes which proposes that fCoA binds to HACL/S and reacts with activated ThDP 1’4’-imino-pirimidine tautomer (carbene), forming fCoA-ThDP ylide as first intermediate (α−carbanion), which then attacks the incoming aldehyde/ketone, resulting in a second ThDP-hydroxyacyl-CoA intermediate, from which the acyl-CoA is released as the last step of the reaction (Figs. 3 and 5). The structures of ApbHACL/S with CoA and fCoA trapped intermediate states serve as examples (Fig. 3c, d). Analysis of electron density near ThDP in the presence of CoA derivatives shows features with implications for the catalytic mechanism. The structure of ApbHACL/S with L-lCoA shows a partly ordered acyl with a clearly visible sulfur atom. Similarly, hCoA is partly ordered in the structure of TbHACL/S. Remarkably, in the structure with fCoA there is an intermediate trapped where there is a covalent bond between C2 of ThDP and carbon of hydroxymethyl moiety that is still attached to the sulfur atom of CoA. This seemingly corresponds to the intermediary α−carbanion state with ThDP covalently attached to hmCoA with an elongated C2-C bond distance. This state is ready to attack the incoming aldehyde/ketone to complete carbon addition. The $\alpha$-carbanion is not very stable, exists in two tautomeric forms and can undergo various chemical transformations^2,16^. Thus, it is quite noteworthy that we were able to capture these states in two structures. However, in several our structures of ApbHACL/S and TbHACL/S with CoA derivatives we observe states where acyl-CoA is no longer connected to ThDP, as the sulfur-carbon bond (S-C2 in acyl-CoA, see Fig. 3a) is broken. These may correspond to post reaction states.

In addition, three structures contain covalent adducts to ThDP (“oxo-ThDP” and “hydroxymethyl-ThDP”) (Fig. 3a, e, f). In these structures “=O” atom or “-C–O” moiety remain attached to C2 of the thiazolium ring. We suggest these “oxo-ThDP” and “hydroxymethyl-ThDP” adducts to be products of off pathway reactions. We propose mechanism of these reactions in Supplementary Fig. S7. To our knowledge, these off-pathway states were never previously reported for ThDP dependent enzymes and provide valuable information for understanding the catalytic mechanism.

CoA remains bound throughout all steps of catalysis, and all the catalytic chemistry occurs at the acyl-sulfur end of the CoA chain. Interactions with the C-terminal covering regions seems essential for this to occur. Our structures provide insight into several of these states without and with bound ligands. Furthermore, ApbHACL/S and TbHACL/S structures of complexes with CoA (six total) determined in this study more than double available structures of related enzymes (PDB ids: 7pt4, 6u9d, 1v5e, 5tma) that reveal an ordered C-terminal region (Fig. 4), however its conformation is very different than for AcHICL/S^14^. This sequence is often referred to as the active site “covering region”, which is essential for ligand binding and catalysis. ApbHACL/S has stronger activity on C2 (acetaldehyde) compared to C1 (formaldehyde) and C3 (propionaldehyde) substrates (Fig. 6, Supplementary Table S4), while TbHACL/S discriminates less harshly between substrate chain length. It suggests that this region of sequence and its dynamics may influence substrate binding and preference. The analysis of these structures sheds light on the conformation of key conserved and variable residues and their potential roles in the ligand binding and catalytic mechanism. The key water molecules were also located in electron density and their role in catalysis is proposed.

The HACL/S enzymes show high structural similarity, including active and ligand binding sites. There is high conservation of residues involved in ThDP and ADP binding (Supplementary Table S3). Analysis of the active site with bound ligands suggested that the protein surface is dynamic and can adjust to accommodate ligands of different lengths explaining substrate promiscuity. Furthermore, flexible side chains of active site residues allow for small conformational changes that facilitate binding of substrates with different carbon atoms length. It is conceivable that residues near the active site may alter surface dynamics and specificity of binding.

In general, clustering variants based on their substrate specificities revealed key residues and regions that determine substrate specificity. However, HACL/S enzymes are substrate promiscuous (Fig. 6, Supplementary Table S4), and such enzymes typically show lower affinities toward substrates. The active site pocket that accommodates ligands is quite hydrophobic and it can accept different acyl chains, although we believe with different affinity. This may explain why we could not obtain crystals for some of the complexes with substrates. Based on the kinetic characterization ApbHACL/S, DhcHACL/S and TbHACL/S are the top three variants that showed the highest catalytic efficiency toward acetaldehyde (Fig. 6). This may explain why only these three variants are fully resolved in complex with ligands when lactyl-CoA was co-crystalized as a substrate. Moreover, ApbHACL/S shows the lowest K_M_ (0.41 mM, Supplementary Table S4) toward acetaldehyde, which could contribute to successful crystallization with formyl-CoA and acetaldehyde, in addition to lactyl-CoA. This could also explain why we were not able to crystallize any complex with gCoA or hCoA as none of the K_M_ values toward their corresponding aldehydes (formaldehyde and propionaldehyde) is below the millimolar range. Adding higher concentration of substrates might address the issue, but the high reactivity of aldehydes and cost of 2-hydroxyacyl-CoA synthesis need to be overcome. Other studies utilize inactive cofactor, 3-deaza-ThDP (dzThDP), instead of ThDP to prevent catalytic turnover, which might help capture the ligand-bound complex better^12,14^.

All our HACL/S structures contain bound ADP in the R domain, which assumes the Rossmann fold nucleotide binding unit. ADP was first observed in the structure of another thiamin diphosphate-dependent enzyme, OXC^11^. It was shown that ADP activates this enzyme, likely by stabilizing its active conformation. The stimulation of decarboxylase activity by ADP may have physiological importance, as oCoA decarboxylation is an essential step in ATP generation. While we observe ADP binding in HACL/S, we could not clarify its functional relevance because kinetic parameters for HACL/S enzymes that we study are relatively small (Supplementary Table S5). This suggests that ADP binding might be an evolutionary artifact.

Structures of both TbHACL/S and ApbHACL/S with and without acyl-CoA indicate that there is no notable difference in main chains and side chains except for the well-ordered C-terminal regions (Supplementary Fig. S5). Comparison of active site structures and ligand binding for CcHACS/L and ApbHACL/S shows relatively small differences but comparison with AcHICL/S shows much larger differences consistent with variations in substrate specificity and kinetic rates (Supplementary Fig. S6).

It is challenging to extract information about kinetic parameters from a structure alone and typically it requires combining structural data with other type of measurements that may be sensitive to substrate preferences and turnover. Potential parameters that may correlate with function and kinetic parameters can be based on structure and sequence analysis (Supplementary Figs. S1 and S6) and may include several additional measurements defining (1) substrate chain length specificity, (2) active site pocket size, volume, shape and dynamics, (3) sequence and order of C-terminal active site covering segment, (4) active site solvent accessibility, (5) conformational changes in the active site upon ligand binding, (6) mutagenesis and careful kinetic measurements. Some of these parameters can be extracted from existing structures like proximity of key catalytic residues, presence of solvent in the active site, possibility for substrate assisted processes, dynamics of C-terminal covering regions, on (binding) and off (dissociation) rates of ligands to the active site. However, these experiments are not trivial as mutagenesis efforts to convert OXC to HACL/S-like activity failed^4,5^. An in-depth analysis of sequence and structural differences of enzyme sub-families could elucidate important regions within the protein structure contributing to catalytic activities and substrate specificities. Application of time-resolved serial crystallography may provide critical information about binding and conformational changes during catalytic events. These efforts should be combined with molecular dynamics calculation to establish pathways for molecular transformations during ligand binding and catalysis and their role in forward and back reactions. Our data suggest that these enzymes are promiscuous, and this should be further investigated by mutagenesis and binding studies to help design more selective variants and expand the range of possible substrates. Obtaining fully resolved crystal structures of additional ligands will allow in-depth characterization of variants with distinct specificities and elucidate the correlation between structure, substrate specificity and the enzyme activity.

## Materials and methods

### Protein expression and purification

DNA sequences containing genes of oxalyl-CoA decarboxylase from *Dehalococcoidia bacterium* (DhcHACL/S), 2-hydroxyacyl-CoA lyase-like protein 1 from *Conidiobolus coronatus NRRL 28638* (CcHACL/S), oxalyl-CoA decarboxylase from *Thermoflexaceae bacterium* (TbHACL/S), oxalyl-CoA decarboxylase from *Chloroflexi bacterium HGW-Chloroflexi-9* (CfhHACL/S) and oxalyl-CoA decarboxylase from *Alphaproteobacteria bacterium* (ApbHACL/S) were synthesized (Twist Bioscience) and cloned to protein expression vector pMCSG53 comprising the N-terminal His_6_-tag plus Tobacco Etch Virus (TEV) protease cleavage site. For protein production plasmids were transformed to *E. coli* BL21-Gold (DE3) strain. Each of 4–6 L Luria-Bertani medium (LB) cell cultures with 150 μg/mL of ampicillin was grown for 3–4 h till OD at 590 nm around 0.8–1.0 at 37 °C. Then, each culture was cooled down to 18 °C and isopropyl-β-D-thiogalactopyranoside (IPTG) was added up to 0.5 mM to induce protein expression. The cultures were incubated while shaking at 200/min overnight (~16 h) at 18 °C. Cells were harvested and suspended in 35 mL of the lysis buffer containing 0.1 M HEPES pH 8.0, 0.5 M NaCl, 20 mM imidazole, 5% (v/v) glycerol, 10 mM β-mercaptoethanol, and frozen at −80 °C for storage until purification.

The expressed proteins were purified by immobilized metal affinity chromatographic steps (IMAC-I and II) followed by size-exclusion chromatography step^17^. Briefly, frozen cells were thawed and lysed using sonication on ice (5 min total time at 139 W power output). Cell debris was removed by centrifugation at 12,500 rpm for 60 min at 4 °C. The proteins were purified either using the vacuum assisted purification system or applied on AKTAxpress system. For DhcHACL/S, CcHACL/S, and ApbHACL/S, the clarified cell lysate was incubated with 3 mL of NiNTA resin (Cytiva, Marlborough, MA, USA) preequilibrated with the lysis buffer for 30 min at 4 °C. The NiNTA resin was then loaded to a column and washed with 15 column volumes (CVs) of lysis buffer, followed by a wash with 15 CVs of lysis buffer supplemented with 50 mM imidazole. The proteins were eluted using lysis buffer supplemented with 500 mM imidazole. For TbHACL/S and CfhHACL/S the crude extract was applied to a 5-mL HisTrap chelating HP column charged with Ni^[+2^ using AKTAxpress (Cytiva). The column was washed with 15 CVs of lysis buffer, eluted with lysis buffer supplemented with 300 mM imidazole and applied to the 100 mL buffer exchange column (two columns HiPrep 26/10 in tandem attached to AKTAxpress, Cytiva), prewashed with the lysis buffer without imidazole. The affinity purification-buffer exchange protocols were used. To the purified protein by IMAC-I, TEV protease was added in 1:200 (TEV protease:protein) ratio and incubated at 4 °C for 16–48 h. The proteins were purified using IMAC-II protocol as described previously^17^. Due to the low yield after IMAC-I step for DhcHACL/S and ApbHACL/S, the His-tag was not cut. The purified protein fractions (either the His-tag removed or not) were pooled and concentrated to about 0.5 μL–2 mL and loaded onto a Superdex 200 10/300 GL (24 mL) or a Superdex 200 16/60 (120 mL) column (Cytiva), (depends on the volume of the concentrated protein) pre-equilibrated in crystallization buffer (20 mM HEPES pH 7.5, 150 mM NaCl and 2 mM DTT). The peak fractions containing the pure proteins were pooled and concentrated to 10–55 mg/mL. The purity of the proteins was verified using SDS-PAGE.

### Protein crystallization and data collection

Crystallization experiments were conducted using the sitting-drop vapor-diffusion method with the help of the Mosquito liquid dispenser (TTP LabTech) in 96-well CrystalQuick plates (Greiner Bio-One). Drops, each containing 0.3–0.4 μL of the protein or protein complex samples and equal volume of crystallization solution (reservoir solution), were allowed to equilibrate at 16 °C. The protein concentrations were 7–15 mg/mL (0.1–0.3 mM) and for the complexes 1–2 mM ThDP, ADP, and acyl-CoAs were added and incubated for at least 30 min at 4 °C or 24 °C before crystallization. Acyl-CoAs used were: fCoA, L- or D-lCoA, and 2hcCoA. All proteins were co-crystallized with ThDP and ADP, but only TbHACL/S and ApbHACL/S were successfully crystallized in the presence of additional ligand acyl-CoAs. Crystallization with several ketone substrates was unsuccessful. Typically, crystals were observed after 1–3 days.

DhcHACL/S with ThDP and ADP crystallized under several conditions and the best diffracting were obtained in a solution containing 0.1 M HEPES at pH 7.5 and 20% (w/v) PEG 8000. The best ApbHACL/S crystals with fCoA were produced under condition of 0.2 M sodium sulfate, 0.1 M BisTris propane at pH 7.5, and 20% (w/v) PEG 3350. The crystals of ApbHACL/S with L- or D-lCoA were obtained under conditions of 0.1 M Tris HCl at pH 7.0, 20% (w/v) PEG 2000 MME, and 0.2 M ammonium phosphate dibasic, 20% (w/v) polyethylene glycol 3350, respectively.

TbHACL/S with ThDP and ADP were crystallized in the condition consisting of sodium/potassium phosphate, 0.1 M BisTris propane pH 6.5, 20% (w/v) PEG 3350, and crystals of TbHACL/S-2hbCoA complex (TbHACL/S-hCoA) containing ThDP, ADP and 2hbCoA produced from 0.1 M MES pH 6.5, 20% (w/v) PEG 10000, and TbHACL/S and ThDP, ADP and fCoA were co-crystallized in 50 mM calcium chloride, 0.1 M MES pH 6.0, 45% (v/v) PEG 200.

All crystals were cryoprotected in their respective reservoir solutions supplemented with 20–30% (v/v) ethylene glycol or glycerol and flash-cooled in liquid nitrogen. For more complete occupancy ligands (ADP, ThDP and acyl-CoAs), the crystals were soaked with these ligands for about 2–4 min before being flash-cooled.

X-ray diffraction experiments were conducted at 100 K at 19-ID beamline at the Structural Biology Center (SBC) and 23-IDB at GM/CA of the Advanced Photon Source (APS) at Argonne National Laboratory using Pilatus3 X 6 M detector (Dectris) and Eiger 16 M detector (Dectris), respectively and at NYX beamline 19-ID of National Synchrotron Light Source 2 (NSLS2) at Brookhaven National Laboratory using Eiger2 9 M XLE detector (Dectris). At SBC and GM/CA, 50 × 50 μm x-ray beam, 10% transmission, 0.5 deg/0.5 s exposures were used and at NYX, 0.2 deg/0.05 s 10 × 10 μm x-ray beam, 20% transmission were used. For highly redundant data 300 to 360 degrees of continuous rotation were collected. Crystals of HACL/S and their complexes diffracted to 1.70–2.70 Å (Supplementary Table S1).

### Synthesis of CoA esters

#### Formyl-CoA

The synthesis was performed following a slight modification of the existing procedures^10,18,19^. First, formyl thiophenol was synthesized. 5.8 (150 mmol) formic acid was added dropwise to 7.1 mL (75 mmol) acetic anhydride and stirred at 25 °C for 2.5 h. Subsequently, 61 µL (0.75 mmol) pyridine, then 5.1 mL (50 mmol) thiophenol were added, and the mixture was stirred overnight. Impurities were distilled away in a rotary evaporator at 50 °C, 25 mbar. The residual mixture was washed in cold brine, dried over MgSO_4_ and distilled at 131 °C, 50–60 mbar to a clear oil. The product was stored under nitrogen at −20 °C. For subsequent formyl-CoA synthesis, 200 mg CoA were dissolved in 2 mL ice-cold 1 M KHCO_3_, pH 8.0 and gassed out by shaking. 0.4 mL formyl-thiophenol was added and the mixture shaken vigorously for 10 min. Cold diethyl ether was used to wash the product, removing phenol and formic acid. Consecutively, the pH was decreased to <4 by addition of HCl and two more ether washes were performed.

#### Synthesis of Glycolyl-CoA

Glycolyl-CoA was synthesized from glycolate and coenzyme A as described previously^20,21^. Briefly, 168 mg of carbonyldiimidazole (CDI) was dissolved in 8 mL of tetrahydrofuran, and 320 mg of glycolate was added. The solution was incubated at room temperature for 20 min while stirring gently. Then, 320 mg of coenzyme A was dissolved in 4 mL of 500 mM NaHCO_3_, and the CDI solution was added. Overnight incubation at 4 °C was followed by the addition of 38 mL of 500 mM NaHCO_3_ and quenching with formic acid to pH 3. The solution was filtered through a 0.45 µm filter and kept under vacuum for 1 h while stirring. Purification of glycolyl-CoA was performed by preparative HPLC-MS in 25 mM ammonium formate at pH 4.2. Fractions containing the product were lyophilized and stored at −80 °C.

#### D- and L-Lactyl-CoA, 2-Hydroxyisobutyryl-CoA

D-lactyl-CoA, L-lactyl-CoA and 2-hydroxyisobutyryl-CoA were synthesized from their respective enantiopure acids using CDI^21^. Acyl-CoAs were separated from free CoA on a preparative Agilent 1260 Infinity HPLC with a Gemini 10 μm NX-C18 110 Å column. The purified CoA esters were flash frozen in liquid N2, lyophilized and stored dry at −20 °C. The masses were verified during purification with mass-spectrometry.

#### Structure determination and refinement

All diffraction data collected data at SBC and NYX were processed by HKL3000^22^ and the data collected at GM/CA (23ID-B) were processed automatically by fast_dp^23^. The highest resolution cut for each data was made based on I/σ (1.00), completeness (65%), and CC_1/2_^24^ (0.4) with exception for the ApbHACL/S-D-lCoA complex which has 0.9, 83.9% and 0.485 for I/σ, completeness, and CC_1/2_, respectively in an effort to include more available reasonable experimental data in refinement. The structure refined well with higher resolution data. The crystals belong to several space groups (see Supplementary Table S1) and contain a dimer (for CfhHACL/S and TbHACL/S complexes with fCoA and hCoA)), two dimers (for CcHACL/S, TbHACL/S and ApbHACL/S complexes with CoA, fCoA, D-lCoA and L-lCoA) or four dimers for DhcHACL/S in the asymmetric unit. All the structures were solved by molecular replacement with the corresponding AlphaFold2^15^ models as search models using Molrep^25^, followed by brief rigid body refinement and initial refinement in Refmac5.5^26^, all implemented in HKL3000. All structures were refined by iterative refinement cycles of manual adjustment using Coot^27^ plus restrained refinement using Phenix (phenix.refine)^28^ until the structures converged to models with reasonable stereochemistry and R/R_free_. The progress of the refinement was carefully monitored with R and R_free_ which were calculated by using randomly selected 5% of reflections from the total unique reflections and these were excluded from all refinement. After each cycle of refinement, the refined structure was checked with Molprobity^29^ and Ramachandran plot^30^. Statistics for all crystal diffraction data collection, processing and structure refinement is shown in Supplementary Table 2. The final refined structures were validated with PDB validation before deposition with PDB ids: of 8vzj, 8vzi, 8vzh, 8vze, 8vzd, 8vzk, 8vzf, 8vzc, 8vza, 8vzb for DhcHACL/S, CcHACL/S, TbHACL/S, TbHACL/S-hCoA, TbHACL/S-fCoA, CfhHACL/S, ApbHACL/S-CoA, ApbHACL/S-fCoA, ApbHACL/S-L-lCoA, Apb-D-lCoA, respectively.

#### Kinetic characterization of HACL/S variants

Different HACL/S variants were cloned into pCDFduet-1 (Novagen), which were then transformed into *E. coli* BL21(DE3) for expression. Overnight cultures of the expression strains were grown in LB with 100 mg/L spectinomycin, which was used to inoculate (1%) 100 mL TB medium supplemented with 50 mg/L spectinomycin in a 500 mL flask. The culture was grown at 30 °C and 250 r.p.m. in an orbital shaker until OD 600 reached 0.4–0.6, at which point expression was induced with 0.05–0.1 mM isopropyl β-d-1-thiogalactopyranoside (IPTG). Then, 24 h after inoculation, cells were collected by centrifugation and stored at −80 °C until needed. The frozen cell pellets were resuspended in 10 mL of cold lysis buffer (50 mM Tris (pH 7.4), 300 mM NaCl, 20 mM imidazole). The mixture was further treated by sonication on ice using a Cole-Parmer ultrasonic processor CPX130 (3 min with 5-s pulse on and a 6-s pulse off cycles, and amplitude set at 30%) and centrifuged at 7500 g for 20 min at 4 °C. The supernatant was applied to a chromatography column containing 3 mL Ni-NTA agarose resin (Qiagen), which had been pre-equilibrated with the lysis buffer. The column was then washed with 15 mL of the lysis buffer and then with 20 mL of wash buffer (50 mM Tris (pH 7.4), 300 mM NaCl, 50 mM imidazole). The His-tagged protein of interest was eluted with 15 mL elution buffer (50 mM NaPi (pH 7.4), 300 mM NaCl, 100 mM imidazole). The eluate was collected and applied to a 10,000 molecular-weight cut-off Amicon ultrafiltration centrifugal device (Millipore), and the concentrate (<300 μL) was washed twice with 4 mL of 50 mM KPi and 10% glycerol (pH 7.4) for desalting. Protein concentrations were calculated using the Bradford Protein Assay (Bio-Rad) according to the manufacturer’s protocol. Purified protein was saved in 20 μL aliquots at −80 °C until needed.

HACL/S kinetic constants were determined by measuring 2-hydroxyacyl-CoA production via base hydrolysis of the CoA thioester and subsequent HPLC analysis to quantify the corresponding carboxylic acid product. Experiments were performed using a 50 μL reaction mixture containing 100 mM KPi pH 6.9, 10 mM MgCl_2_, 0.15 mM TPP and 0.1–1 μM HACL/S. To determine kinetic constants for C1–C3 aldehyde substrates, a constant in-situ formyl-CoA generation method was applied by using 2 μM CaAbft^6^ to catalyze 20 mM formate with 2 mM acetyl-CoA as the CoA donor. The room temperature reactions were initiated by adding different concentrations of aldehyde substrates. Aldehyde and HACL/S enzyme concentrations were varied for each HACL/S to achieve saturating conditions and initial velocity was determined for 8 different aldehyde concentrations. Reaction samples were taken at 2, 3, 5, 7, and 10 min and quenched by the addition of 5 μL 10 M NaOH for 45 min. After that 5 μL of 10 N sulfuric acid was added and the samples were analyzed by HPLC for the determination of the initial reaction rates. Data was analyzed in GraphPad Prism using a Michalis Menten fit (Fig. 6, Supplementary Table S4).

#### Computer software

All diffraction data collected data at SBC and NYX were processed by HKL3000^22^ and the data collected at GM/CA (23ID-B) were processed automatically by fast_dp^23^. All the structures were solved by molecular replacement with the corresponding AlphaFold2^15^ models as search models using Molrep^25^, followed by brief rigid body refinement and initial refinement in Refmac5.5^26^, all implemented in HKL3000. All structures were refined by Coot^27^ plus Phenix (phenix.refine)^28^. The refined structures were checked with Molprobity^29^ and Ramachandran plot^30^. The final refined structures were validated with PDB validation (https://deposit-2.wwpdb.org/deposition).

### Statistics and reproducibility

The results included in this manuscript can be reproduced by following protocols and using materials described in Material and Methods.

### Reporting summary

Further information on research design is available in the Nature Portfolio Reporting Summary linked to this article.

### Supplementary information

Supplementary Information

Reporting Summary

## Data Availability

The structural datasets generated during the current study are available in the Protein Data Bank repository (https://www.rcsb.org/) under accession codes: 8vzj, 8vzi, 8vzh, 8vze, 8vzd, 8vzk, 8vzf, 8vzc, 8vza, 8vzb. Plasmids for protein expression are available upon request. All other data generated during the current study including the raw kinetic and biophysical data are available upon request.
