# Supplementary material for: Revealing reaction intermediates in one-carbon elongation by thiamine diphosphate/CoA-dependent enzyme family
Source: Commun Chem. 2024 Jul 21;7:160. doi: 10.1038/s42004-024-01242-y (PMC11271303; doi:10.1038/s42004-024-01242-y)
Supplement: Supplementary file 1 — Supplementary Information [file 42004_2024_1242_MOESM1_ESM.pdf]

Supplementary figures

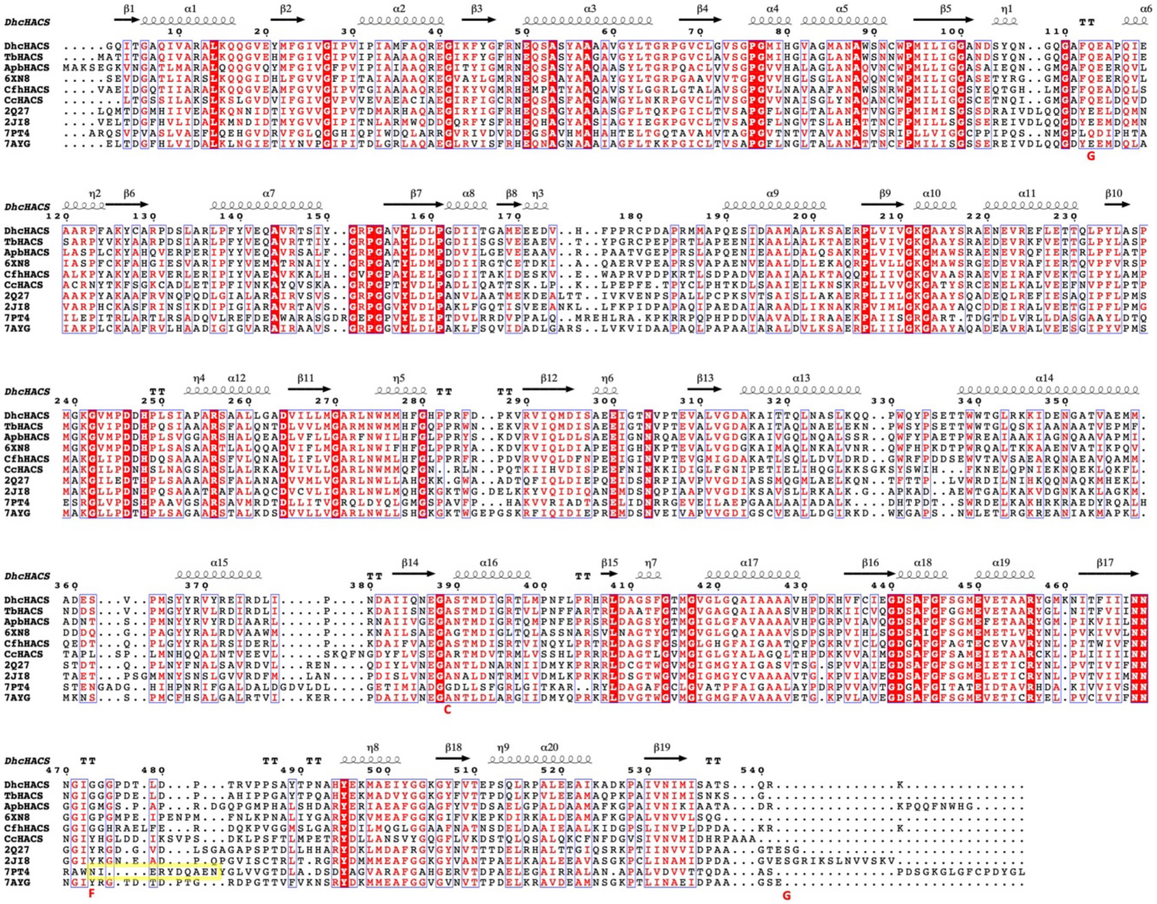

Supplementary Figure S1. Sequence alignment of HACL/S including RuHACL/S, OXC and AcHICL/S sub-families. Y497F, S568G, E135G, and A415C mutations from MeOXC (PDB id: 7ayg) to MeOXC4 (PDB id: 7b2e)[1] are indicated in red just below 7ayg sequence. Yellow box indicates the α-helix in AcHICL/S (PDB id: 7pt4) distinct from other enzymes.

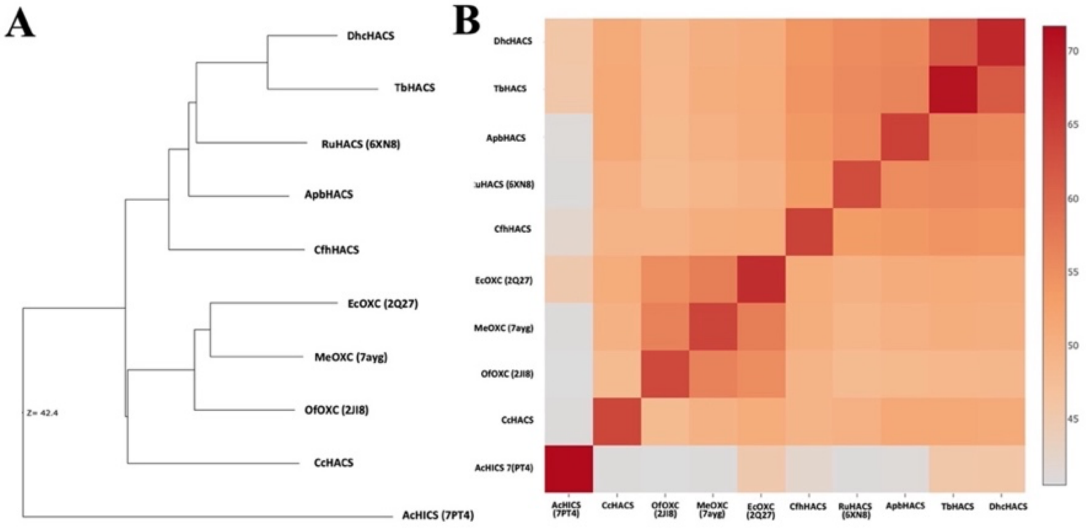

Supplementary Figure S2. Structural similarity tree (A) and dendrogram (B). The dendrogram is derived by average linkage clustering of the structural similarity matrix (Dali Z-scores)[2].

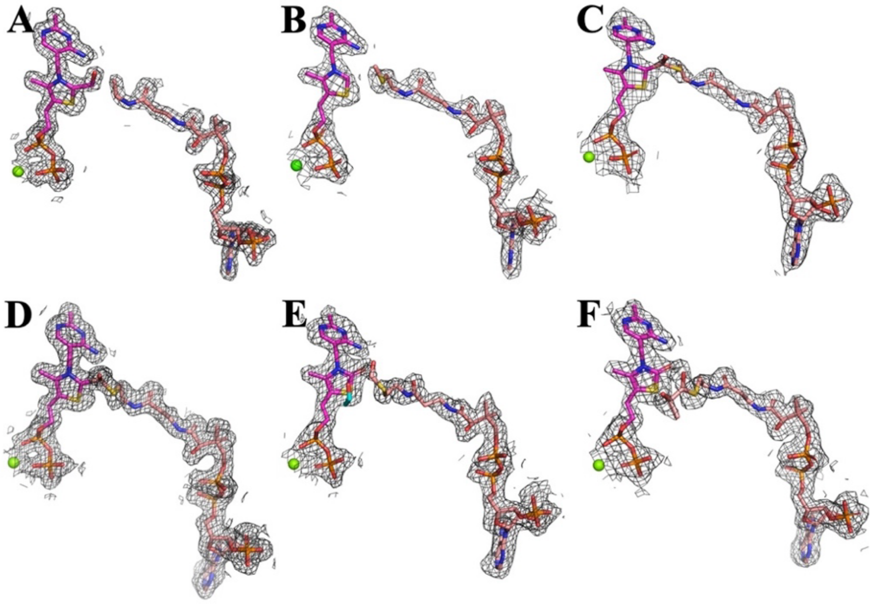

Supplementary Figure S3. Electron density for ThDP and acyl-CoA molecules found in the TbHACL/S-fCoA (A), TbHACL/S-hCoA (B), ApbHACL/S-CoA (C), ApbHACL/S-fCoA (D), ApbHACL/S-L-lCoA (E), and ApbHACL/S-D-lCoA (F) complexes. Magnesium ion is shown as green sphere. The electron density (composite annealed map, 2DFo-mFc) for the ligands is contoured at 1.0 σ level.

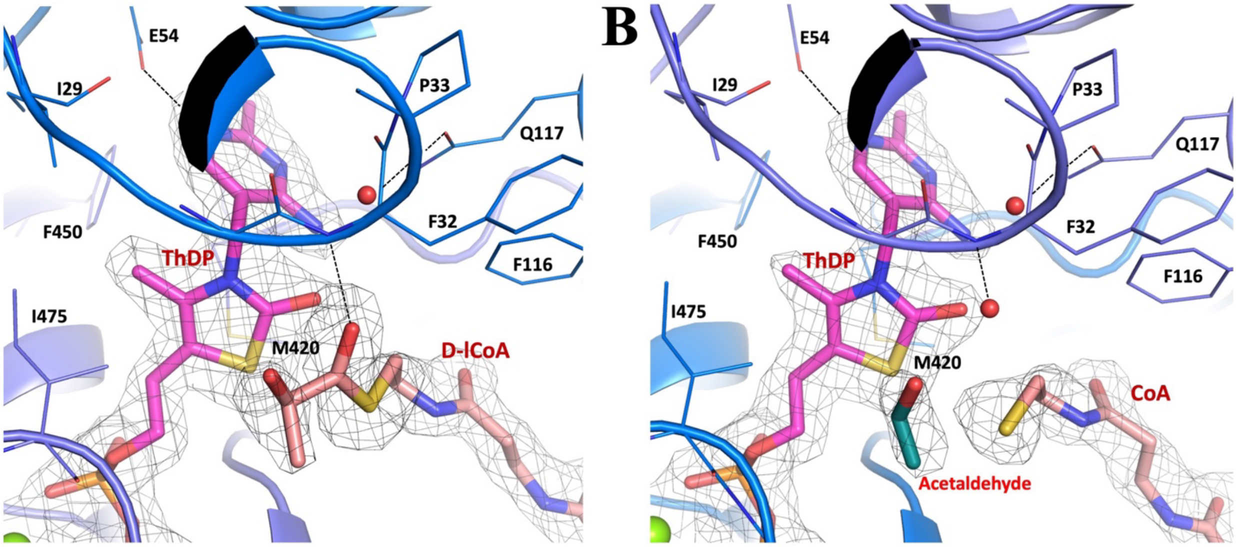

Supplementary Figure S4. Active sites of ApbHACL/S-D-lCoA contain two different species of acyl-CoA, D-lCoA (A) and hydrolyzed to acyl-CoA plus acetaldehyde (B). ThDP and acyl-CoA molecules shown in magenta and orange sticks, respectively and acetaldehyde in dark green. The electron density (composite annealed map, 2DFo-mFc) for the ligands is contoured at 1.0 σ level.

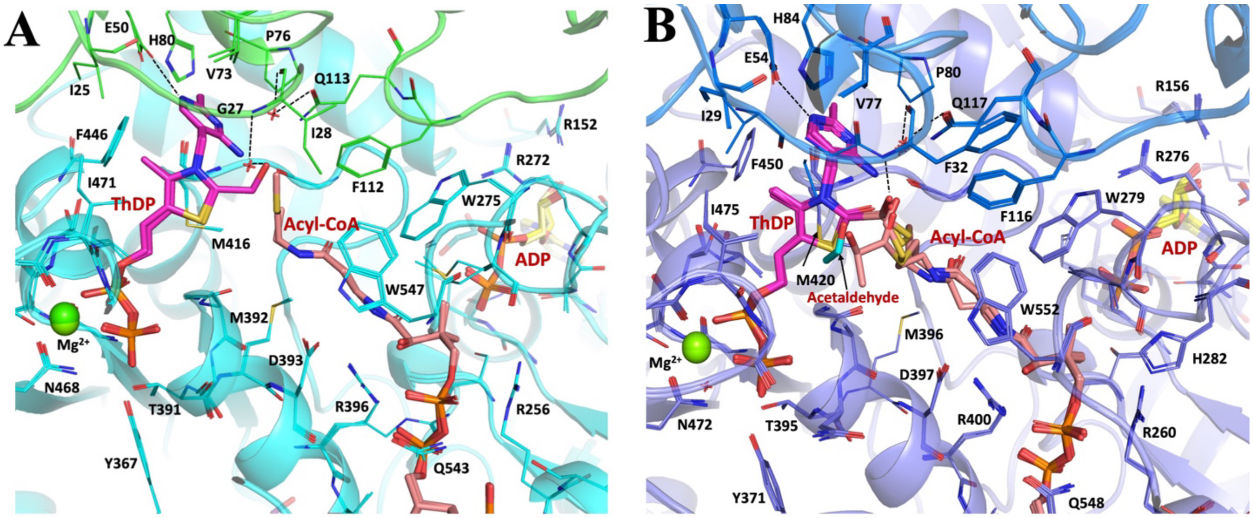

Supplementary Figure S5. Structure of active sites of TbHACL/S complex (A) and ApbHACL/S complex (B) showing interaction of enzymes with ThDP and acyl-CoAs. TbHACL/S in cyan and green cartoon drawing, ApbHACL/S in fade blue and dark blue, ThDP in magenta sticks and acyl-CoAs in orange.

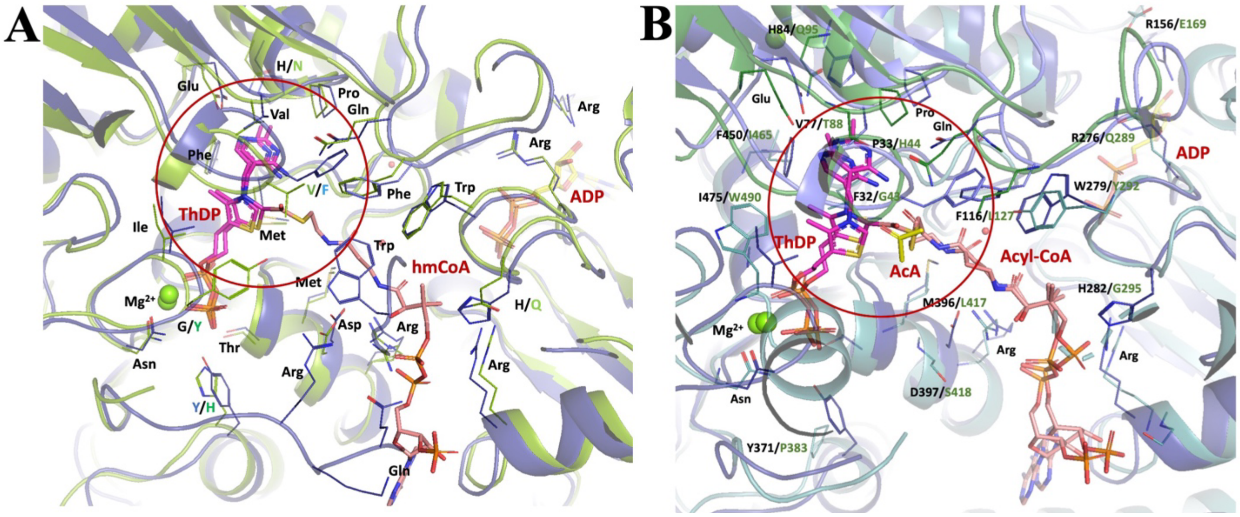

Supplementary Figure S6. Differences between active sites in enzymes (A) CcHACL/S (light green) and ApbHACL/S (dark blue) and (B) ApbHACL/S (dark blue) and AcHICL/S (pale green). Active sites in circles. ThDP in magenta sticks and acyl-CoAs in orange sticks.

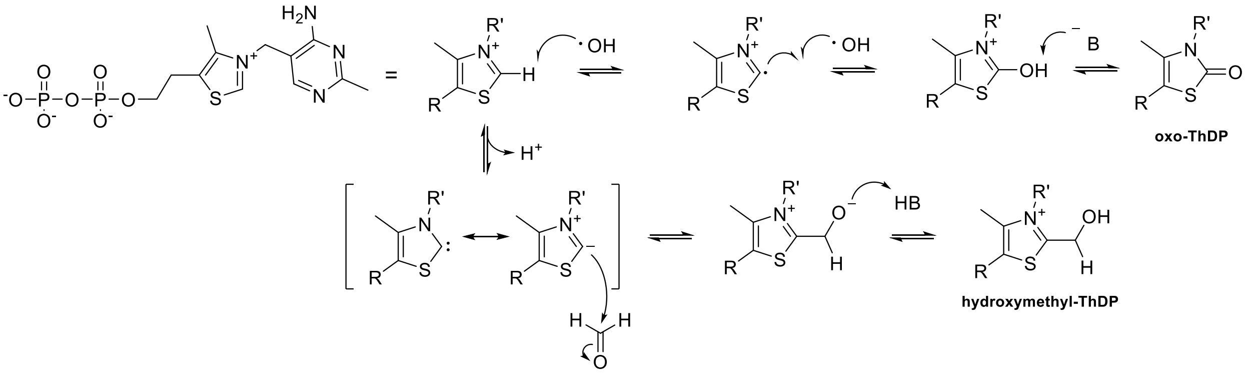

Supplementary Figure S7. Possible mechanisms for generation of “oxo-ThDP” and “hydroxymethyl-ThDP” via a ROS mechanism (an example is provided with hydroxyl radical) or an attack of formaldehyde, respectively.

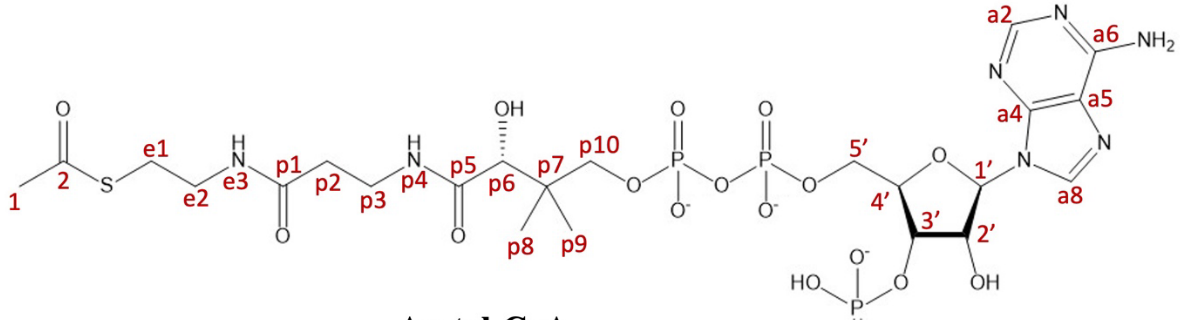

Acetyl-CoA

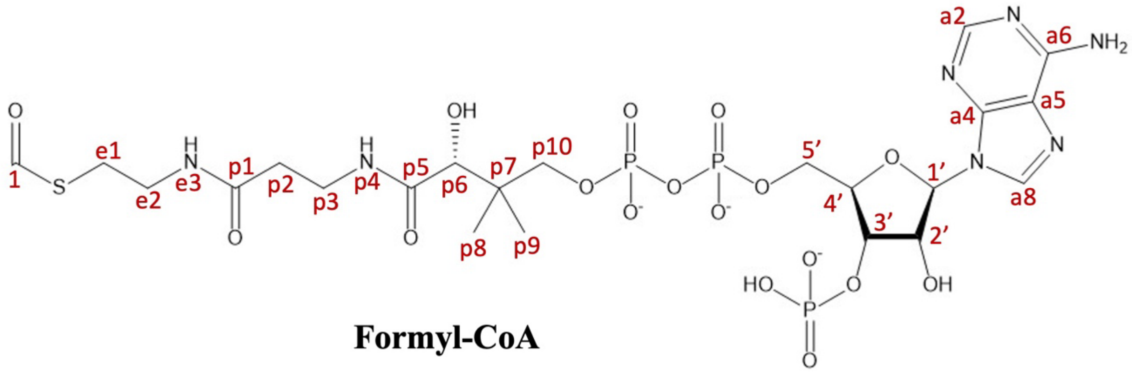

Formyl-CoA

Supplementary Figure S8. Carbon atom numbering schemes for acetyl-CoA and formyl-CoA

Supplementary tables

Supplementary Table S1. Crystal data and refinement statistics

	DhcHACL/S	*CcHACL/S*	*TbHACL/S*	*CfhHACL/S*
Data Collection	19-ID SBC	19-ID SBC	19-ID SBC	19-ID SBC
Space group	*C*2	$P2_1$	$P2_12_12_1$	$P3_121$
Cell dimensions				
a, b, c (Å)<br>*α, β, γ (°)*	324.22,77.84, 229.55, $\alpha=\gamma=90$, $\beta=134.5$	64.74, 179.29, 96.83, $\alpha=\gamma=90$, $\beta=108.2$	120.59, 134.01, 173.39, $\alpha=\beta=\gamma=90$	$a=b=$ 111.33, $c=$193.16, $\alpha=\beta=90$, $\gamma=120$
Resolution range (Å)[a]	2.70-50.00 (2.70-2.75)	1.85-50.00 (1.85-1.88)	2.25-50.00 (2.25-2.29)	2.65-50.00 (2.65.2.70)
Unique reflections[a]	112,373 (5,449)	167,273 (6,685)	133,571 (6,589)	39,660 (1,349)
R-merge[b]	0.175 (1.686)	0.107 (0.818)	0.159 (1.196)	0.162 (0.913)
Mean I/σ(I)	10.1 (1.1)	14.7 (1.3)	10.3 (1.6)	13.0 (1.0)
$CC_{1/2}$[c]	0.996 (0.423)	0.996 (0.791)	0.983 (0.659)	1.0 (0.837)
Completeness (%)	99.7 (97.8)	94.1 (76.1)	99.9 (100)	97.1 (68.4)
Redundancy	8.5 (7.0)	7.8 (5.8)	5.2 (5.0)	14.2 (8.7)
Wilson B-factor (Å²)	66.0	22.4	27.6	59.2
Refinement				
Resolution range (Å)	2.70-40.53 (2.70-2.73)	1.85-44.82 (1.85-1.87)	2.25-43.39 (2.25-2.27)	2.65-48.23 (2.65-2.72)
Reflections work/test	106,761/5,492	158,800/8,345	126,194/6,707	37,562/1,913
R_{work}/R_{free}	0.194/0.235	0.156/0.196	0.160/0.195	0.208/0.244
Number atoms	33287	18514	17416	8326
Protein	32822	16813	16299	8195
Ligand/ion	460	298	297	127
Water	5	1403	820	4
Protein residues	4317	2234	2175	1092
RMSD (bonds) (Å)	0.002	0.006	0.004	0.002
RMSD (angles) (°)	0.513	0.876	0.690	0.481
Ramachandran favored[c] *(%)*	96.96	97.72	98.23	97.22
allowed (%)	3.01	2.19	1.77	2.59
outliers (%)	0.02	0.09	0.0	0.19

Rotamer outliers (%)[d]	1.81	0.87	0.72	2.67
Clashscore	4.73	2.64	1.64	3.25
Average B-factor (Å^2)	77.2	29.7	32.5	69.6
Protein	77.3	29.6	32.5	69.8
Ligand/ion	66.3	26.5	32.0	62.3
Water	74.2	33.6	33.0	54.6
Number of TLS groups	53	31	23	12
PDB ID	8vzj	8vzi	8vzh	8vzk

	TbHACL/S-fCoA	*TbHACL/S-hCoA*	*ApbHACL/S-CoA*	*ApbHACL/S-fCoA*	*ApbHACL/S-L-lCoA*	*ApbHACL/S-D-lCoA*
Data Collection	23-IDB GMCA	19-ID SBC	19-ID SBC	19-ID SBC	19-ID SBC	23-IDB GMCA
Space group	$C222_1$	$C222_1$	$P2_12_12_1$	*P*1	*P*1	$P2_12_12_1$
Cell dimensions						
a, b, c (Å), *α, β, γ (°)*	100.17, 182.51, 123.92, $\alpha=\beta=\gamma=90$	100.62, 182.77, 123.84, $\alpha=\beta=\gamma=90$	69.80, 178.13, 196.48, $\alpha=\beta=\gamma=90$	69.08, 102.44, 103.98, 113.8, 96.8, 105.5	69.41, 102.68, 104.34, 114.1, 96.7, 105.2	69.71, 178.00, 196.11, $\alpha=\beta=\gamma=90$
Resolution range (Å)[a]	2.20-50.00 (2.20-2.24)	1.70-50.00 (1.70-1.73)	2.36-50.00 (2.36-2.39)	1.72-50.00 (1.72-1.76)	2.05-50.00 (2.05-2.09)	1.98-50.00 (1.98-2.08)
Unique reflections[a]	56,818 (2,390)	123,996 (5,095)	99,068 (4,335)	23,8150 (1,487)	144,733 (5,583)	165,964 (20,426)
R-merge[b]	0.233 (0.678)	0.188 (0.850)	0.263 (1.126)	0.052 (0.338)	0.189 (0.671)	0.275 (2.039)
Mean I/σ(I)	6.8 (1.0)	14.0 (1.2)	6.2 (1.1)	7.2 (1.6)	7.9 (1.4)	7.8 (0.9)
$CC_{1/2}$[c]	0.977 (0.576)	0.984 (0.506)	0.960 (0.548)	0.996 (0.806)	0.928 (0.490)	0.995 (0.435)
Completeness (%)	98.1 (83.7)	98.3 (81.4)	97.2 (85.6)	92.3 (59.9)	94.9 (73.2)	97.3 (83.9)
Redundancy	8.2 (3.0)	8.0 (3.8)	5.8 (4.9)	2.7 (2.6)	3.5 (2.7)	12.0 (7.2)
Wilson B-factor (Å^2)	21.6	14.9	32.7	18.4	25.4	25.7
Refinement						
Resolution range (Å)	2.20-37.41 (2.20-2.24)	1.70-48.04 (1.70-1.71)	47.35-2.36 (2.36-2.38)	1.72-38.63 (1.72-1.74)	2.05-49.52 (2.05-2.08)	1.98-47.27 (1.98-2.00)
Reflections work/test	53,986/2,797	117,122/6,044	93,957/4,893	226,339/11,600	137,400/7,055	156,814/8,175
R_{work}/R_{free}	0.198/0.234	0.145/0.175	0.208/0.246	0.160/0.199	0.209/0.243	0.182/0.219
Number atoms	9071	9574	17547	18812	17923	18742
Protein	8313	8463	16756	16734	16695	16745
Ligand/ion	245	173	454	344	438	514

Water	513	938	337	2078	790	1483
Protein residues	1103	1135	2226	2228	2219	2227
RMSD (bonds) (Å)	0.002	0.011	0.002	0.008	0.002	0.002
RMSD (angles) (°)	0.561	1.138	0.696	1.048	0.666	0.692
Ramachandran favored[c] (%)	97.81	98.44	97.25	98.05	97.73	97.69
allowed (%)	2.19	1.56	2.75	1.95	2.23	2.31
outliers (%)	0.00	0.00	0.0	0.00	0.05	0.00
Rotamer outliers (%)[d]	1.53	0.82	1.08	0.84	0.85	0.60
Clashscore	2.01	1.06	2.62	1.21	2.07	1.35
Average B-factor (Å^2)	23.3	17.5	36.2	22.7	25.1	26.6
Protein	23.3	16.6	36.2	21.7	25.1	26.2
Ligand/ion	23.6	20.0	39.3	26.6	24.1	30.7
Water	23.4	25.7	32.3	30.5	26.7	30.4
Number of TLS groups	13	10	24	23	27	30
PDB ID	8vzd	8vze	8vzf	8vzc	8vza	8vzb

[a] Values in parentheses correspond to the highest resolution shell.

[b] Rmerge = ΣhΣj|Ihj–<Ih>|/ΣhΣjIhj, where Ihj is the intensity of observation j of reflection h.

[c] As defined by Karplus and Diederichs[3].

[d]As defined by Molprobity[4].

Supplementary Table S2: Pairwise Comparisons of RMSD and sequence identity.

The numbers are shown in the following order: pairwise sequence identities (%), RMSDs of /540 superimposed C⍰ atom positions of active site residues/residues binding to ThDP, ADP, or acyl-CoA/residues binding to ThDP or acyl-CoA/residues binding to ThDP. The residues in active sites, binding to ThDP and ADP and used for calculations are listed in Supplementary Table S3.

Name	**CcHACL/S**	**TbHACL/S**	**CfhHACL/S**	**ApbHACL/S**	**RuHACL/S**	**OfOXC (2ji8)**	**EcOXC (2q27)**	**MeOXC (7ayg)**	**AcHICL/ S (7pt4)**
DhcHACL/S	43.2/1.33<br>0.56/0.55<br>0.51	79.0/0.47<br>0.23/0.29<br>0.28	51.5/1.00<br>0.54/0.51<br>0.52	61.7/0.74<br>0.35/0.42<br>0.40	53.7/0.87<br>0.48/0.40<br>0.37	40.4/1.57<br>0.60/0.62<br>0.63	41.8/1.23<br>0.65/0.62<br>0.66	41.4/1.31<br>0.740.69<br>0.75	25.5/1.92<br>1.49/1.45<br>1.47
CcHACL/S		41.7/1.28<br>0.62/0.49<br>0.44	39.0/1.53<br>0.80/0.61<br>0.58	42.2/1.25<br>0.57/0.55<br>0.49	40.4/1.25<br>0.63/0.56<br>0.49	40.2/1.55<br>0.57/0.72<br>0.72	41.9/1.36<br>0.56/0.58<br>0.55	37.8/1.48<br>0.58/0.80<br>0.79	23.5/1.71<br>1.37/1.35<br>1.38
TbHACL/S			52.5/0.99<br>0.54/0.44<br>0.44	60.7/0.70<br>0.36/0.29<br>0.29	55.3/0.78<br>0.45/0.28<br>0.25	40.0/1.60<br>0.67/0.67<br>0.67	41.1/1.24<br>0.70/0.55<br>0.55	41.2/1.35<br>0.76/0.78<br>0.76	24.5/1.92<br>1.52/135<br>1.36
CfhHACL/S				51.1/0.99<br>0.58/0.52<br>0.53	47.9/1.23<br>0.67/0.41<br>0.35	38.1/1.44<br>0.83/0.78<br>0.79	42.5/1.33<br>0.84/0.73<br>0.74	39.8/1.38<br>0.92/0.90<br>0.90	29.6/1.85<br>1.46/1.35<br>1.34
ApbHACL/S					62.1/0.73<br>0.49/0.39<br>0.37	37.7/1.49<br>0.65/0.69<br>0.69	39.6/1.24<br>0.65/0.59<br>0.59	39.8/1.40<br>0.73/0.79<br>0.77	29.6/1.82<br>1.49/1.41<br>1.40
RuHACL/S (6xn8)						38.6/1.53<br>0.71/0.77<br>0.75	41.2/1.33<br>0.78/0.67<br>0.67	39.1/1.51<br>0.85/0.88<br>0.85	27.3/1.76<br>1.49/1.46<br>1.45
OfOXC (2ji8)							55.7/0.82<br>0.43/0.42<br>0.43	62.0/0.79<br>0.35/0.38<br>0.37	22.9/1.85<br>1.32/1.27<br>1.29
EcOXC (2q27)								61.3/0.74<br>0.43/0.43<br>0.42	24.6/1.85<br>1.26/1.13<br>1.14
MeOXC (7ayg)									26.2/1.88<br>1.27/1.15<br>1.17

Supplementary Table S3: Residues involved in interacting with ThDP, acyl-CoA, and ADP

Name	Catalytic site-ThDP	Pyrophosphate-ThDP	Acyl-CoA	ADP
DhcHACS	G27-P29, E50, F112-Q113, G414, M416,	A389-T391, Y367, I472-G473, D441-A443, N469,	R256, R396,	R152, R272, K212, D295-I296, S237, A315,
CcHACS	G29-P31, E52, F114-Q115, G416, M418,	A391-T393, H365, I473-Y474, D443-A445, N470),	R256, R398,	R154, R272, K212, D293-I294, T237, I313,
TbHACS	G27-P29, E50, F112-Q113, G414, M416,	A389-T391, Y367, I471-G472, D441-A443, (N468	R256, R396,	R152, R272, K212, D295-I296, S237), A315,
CfhHACS	G34-P36, E57, F119-Q120, G421, M423,	A396-T398, Y374, I478-G479, D448-A450, N475	R263, R403,	V159, R279, K219, D302-F303, M244, A322,
ApbHASL	G31-P33, E54, F116-Q117, G418, M420,	A393-T395, I475-G476; Y371, D445-A447, N472),	R260, R400,	R156, R276, K216, D299-L300, T241, G319,
RuHACS (6xn8)	G27-P29, E50, F112-Q113, G414, M416,	A389-T391, Y367, I471-G472; D441-A443, N468),	R272, L396,	R152, R256, K212, D295-I296, S237, G315,
OfOXC (2ji8)	G33-P35, E56, Y120-E121, G426, M428,	A401-A403, Y377, I482-Y483; D452-A454, N479),	R282, R408,	R160, R266, K222, D306-I307, M247, I326,
AcHICS (7pt4)	G42-H44, E65, L127-Q128, G433, L435,	G410- L412, P383, W490-N491, D460-A462, N487),	R273, R417,	E169, R288, Q289, R230, D311-D312, T254, P331,
EcHACS (2q27)	G31-P33, E54, Y118-E119, M421, M423	A396-T398, Y372, I477-Y478, D447-A449, N470),	R264, R403,	R158, R280, K220, D302-I303, M245, I322,

Residues highlighted in green are from the second subunit (protomer)

Supplementary Table S4. Kinetic parameters of HACL/S variants determined for HACL/S selected for structural analysis.

Substrate	$k_{cat,app}$ (s^{-1})	$K_{m,app}$ (mM)	$k_{cat,app}/K_{m,app}$ (mM^{-1} s^{-1})
**RuHACL/S**			
**Formaldehyde**	3.72 ± 0.66	7.40 ± 0.97	5.03×10^2
**Acetaldehyde**	3.63 ± 0.58	4.11 ± 0.41	8.82×10^2
**Propionaldehyde**	3.36 ± 0.17	17.17 ± 1.11	1.96×10^2
**ApbHACL/S**			
**Formaldehyde**	11.27 ± 0.31	2.028 ± 0.34	5.56×10^3
**Acetaldehyde**	34.89 ± 2.30	0.41 ± 0.06	8.51×10^4
**Propionaldehyde**	10.17 ± 1.09	1.65 ± 0.12	6.17×10^3
**DhcHACL/S**			
**Formaldehyde**	12.27 ± 1.11	2.12 ± 0.37	5.79×10^3
**Acetaldehyde**	41.35 ± 4.00	0.97 ± 0.10	4.26×10^4
**Propionaldehyde**	20.58 ± 1.88	1.071 ± 0.10	1.92×10^4
**CcHACL/S**			
**Formaldehyde**	N.D	N.D.	N.D.
**Acetaldehyde**	26.71 ± 4.23	4.76 ± 0.58	5.61×10^3
**Propionaldehyde**	31.65 ± 2.46	3.497 ± 0.33	9.05×10^3
**TbHACL/S**			
**Formaldehyde**	9.681 ± 0.31	3.73 ± 0.12	2.60×10^3
**Acetaldehyde**	28.63 ± 3.70	2.94 ± 0.49	9.73×10^3
**Propionaldehyde**	24.91 ± 2.55	2.21 ± 0.31	1.13×10^4
**CoHACL/S**			

**Formaldehyde**	10.63 ± 1.47	9.47 ± 1.62	1.12×10^3
**Acetaldehyde**	58.43 ± 9.11	3.80 ± 0.29	1.53×10^4
**Propionaldehyde**	13.08 ± 1.89	1.37 ± 0.22	9.57×10^2
**CfhHACL/S**			
**Formaldehyde**	16.60 ± 1.60	2.47 ± 0.33	6.73×10^3
**Acetaldehyde**	59.23 ± 5.47	8.83 ± 1.09	6.70×10^3
**Propionaldehyde**	5.69 ± 0.63	3.61 ± 0.42	1.58×10^3

Supplementary Table S5. Kinetic parameters of ApbHACL/S on acetaldehyde with and without 0.5 mM ADP. While apparent k_{cat} and catalytic efficiency (k_{cat}/K_M) are slightly higher with ADP, the differences are not statistically significant ($p = 0.55$).

Substrate	ADP (mM)	$k_{cat,app}$ (s^{-1})	$K_{m,app}$ (mM)	$k_{cat,app}/K_{m,app}$ (mM^{-1} s^{-1})
**Acetaldehyde**	0.5	34.51 ± 4.02	0.49 ± 0.10	7.04×10^4
**Acetaldehyde**	0	29.01 ±6.51	0.51 ± 0.11	5.69×10^4

Supplementary References

1. Nattermann, M. *et al.* Engineering a Highly Efficient Carboligase for Synthetic One-Carbon Metabolism. *ACS Catalysis* **11**, 5396-5404, doi:10.1021/acscatal.1c01237 (2021).
2. Holm, L., Laiho, A., Törönen, P. & Salgado, M. DALI shines a light on remote homologs: One hundred discoveries. *Protein Science* **32**, e4519, doi:https://doi.org/10.1002/pro.4519 (2023).
3. Karplus, P. A. & Diederichs, K. Linking Crystallographic Model and Data Quality. *Science* **336**, 1030-1033, doi:10.1126/science.1218231 (2012).
4. Davis, I. W., Murray, L. W., Richardson, J. S. & Richardson, D. C. MOLPROBITY: structure validation and all-atom contact analysis for nucleic acids and their complexes. *Nucleic Acids Res* **32**, W615-619, doi:10.1093/nar/gkh39832/suppl_2/W615 [pii] (2004).
