# Supplementary material for: Revealing reaction intermediates in one-carbon elongation by thiamine diphosphate/CoA-dependent enzyme family
Source: Commun Chem. 2024 Jul 21;7:160. doi: 10.1038/s42004-024-01242-y (PMC11271303; doi:10.1038/s42004-024-01242-y)
Supplement: Supplementary file 2 — Reporting Summary [file 42004_2024_1242_MOESM2_ESM.pdf]

Corresponding author(s): Ramon Gonzalez, Karolina Michalska, Andrzej Joachimiak, Yasuo Yoshikuni

Last updated by author(s): Jul 2, 2024

Reporting Summary

Nature Portfolio wishes to improve the reproducibility of the work that we publish. This form provides structure for consistency and transparency in reporting. For further information on Nature Portfolio policies, see our Editorial Policies and the Editorial Policy Checklist.

Statistics

For all statistical analyses, confirm that the following items are present in the figure legend, table legend, main text, or Methods section.

n/a	Confirmed	
☒	☐	The exact sample size (*n*) for each experimental group/condition, given as a discrete number and unit of measurement
☒	☐	A statement on whether measurements were taken from distinct samples or whether the same sample was measured repeatedly
☒	☐	The statistical test(s) used AND whether they are one- or two-sided<br>*Only common tests should be described solely by name; describe more complex techniques in the Methods section.*
☒	☐	A description of all covariates tested
☒	☐	A description of any assumptions or corrections, such as tests of normality and adjustment for multiple comparisons
☒	☐	A full description of the statistical parameters including central tendency (e.g. means) or other basic estimates (e.g. regression coefficient) AND variation (e.g. standard deviation) or associated estimates of uncertainty (e.g. confidence intervals)
☒	☐	For null hypothesis testing, the test statistic (e.g. *F*, *t*, *r*) with confidence intervals, effect sizes, degrees of freedom and *P* value noted<br>*Give P values as exact values whenever suitable.*
☒	☐	For Bayesian analysis, information on the choice of priors and Markov chain Monte Carlo settings
☒	☐	For hierarchical and complex designs, identification of the appropriate level for tests and full reporting of outcomes
☒	☐	Estimates of effect sizes (e.g. Cohen's *d*, Pearson's *r*), indicating how they were calculated

Our web collection on statistics for biologists contains articles on many of the points above.

Software and code

Policy information about availability of computer code

Data collection	The X-ray diffraction data were recorded at 100K using two beamlines at the Advanced Photon Source at Argonne National Laboratory: the SBC 19-ID-D beamline on the PILATUS3 X 6M detector using SBCcollect software and at GM/CA 23-ID-B on the Eiger 16M detector using Dectris software. The X-ray beam was 50 x 50 [illegible]m with 10% transmission and 0.5 deg/0.5 sec exposures. The X-ray diffraction data collection was recorded also at National Synchrotron Light Source 2 at Brookhaven National Laboratory at NYX beamline 19-ID using Eiger2 9M XLE detector using Dectris software. The X-ray beam was 10 x 10 [illegible]m with 20% transmission and 0.2 deg/0.05 sec exposures. For highly redundant data 300–- 360 degrees of continuous rotation were collected.
Data analysis	X-ray diffraction data collected at the SBC 19-ID and NYX 19-ID were indexed and integrated using HKL3000 and the data collected at GM/CA (23-ID-B) were processed automatically by fast_dp software. All the structures were solved by molecular replacement with the corresponding AlphaFold2 models as search models using Molrep, followed by brief rigid body refinement and initial refinement in Refmac5.5, all implemented in HKL3000. All structures were refined by iterative refinement cycles of manual adjustment using Coot plus restrained refinement using Phenix (phenix.refine) until the structures converged to models with reasonable stereochemistry and R/Rfree. The progress of the refinement was carefully monitored with R and Rfree which were calculated by using randomly selected 5% of reflections from the total unique reflections and these were excluded from all refinement. After each cycle of refinement, the refined structure stereochemistry was checked with Molprobity and Ramachandran plot. Water molecules were generated using the tool find waters in Coot and inspected manually in Coot. The final refined structures were validated with PDB validation server.

For manuscripts utilizing custom algorithms or software that are central to the research but not yet described in published literature, software must be made available to editors and reviewers. We strongly encourage code deposition in a community repository (e.g. GitHub). See the Nature Portfolio guidelines for submitting code & software for further information.

Data

Policy information about availability of data

All manuscripts must include a data availability statement. This statement should provide the following information, where applicable:

- Accession codes, unique identifiers, or web links for publicly available datasets
- A description of any restrictions on data availability
- For clinical datasets or third party data, please ensure that the statement adheres to our policy

The structural datasets generated during the current study are available in the Protein Data Bank repository (https://www.rcsb.org/) under accession codes: 8vzj, 8vzi, 8vzh, 8vze, 8vzd, 8vzk, 8vzf, 8vzc, 8vza, 8vzb. Plasmids for protein expression are available upon request. All other data generated during the current study including the raw kinetic and biophysical data are available upon request.

Research involving human participants, their data, or biological material

Policy information about studies with human participants or human data. See also policy information about sex, gender (identity/presentation), and sexual orientation and race, ethnicity and racism.

Reporting on sex and gender	N/A
Reporting on race, ethnicity, or other socially relevant groupings	N/A
Population characteristics	N/A
Recruitment	N/A
Ethics oversight	N/A

Note that full information on the approval of the study protocol must also be provided in the manuscript.

Field-specific reporting

Please select the one below that is the best fit for your research. If you are not sure, read the appropriate sections before making your selection.

☒ Life sciences ☐ Behavioural & social sciences ☐ Ecological, evolutionary & environmental sciences

For a reference copy of the document with all sections, see nature.com/documents/nr-reporting-summary-flat.pdf

Life sciences study design

All studies must disclose on these points even when the disclosure is negative.

Sample size	Crystals were characterized by single crystals cryo-crystallography. The best diffracting crystals were used to collect complete data set for each protein sample.
Data exclusions	All collected diffraction images were included.
Replication	Ten crystal structures were determined at cryogenic temperature.
Randomization	Throughout refinement, the 5% of randomly selected reflections were kept out throughout from the refinement (in PHENIX protocol) to assess quality
Blinding	N/A

Reporting for specific materials, systems and methods

We require information from authors about some types of materials, experimental systems and methods used in many studies. Here, indicate whether each material, system or method listed is relevant to your study. If you are not sure if a list item applies to your research, read the appropriate section before selecting a response.

Materials & experimental systems

n/a	Involved in the study
☒	☐ Antibodies
☒	☐ Eukaryotic cell lines
☒	☐ Palaeontology and archaeology
☒	☐ Animals and other organisms
☒	☐ Clinical data
☒	☐ Dual use research of concern
☒	☐ Plants

Methods

n/a	Involved in the study
☒	☐ ChIP-seq
☒	☐ Flow cytometry
☒	☐ MRI-based neuroimaging

Plants

Seed stocks	N/A
Novel plant genotypes	N/A
Authentication	N/A
